# Quantized chiral anomaly materials cloak

**DOI:** 10.1038/s41598-017-03587-y

**Published:** 2017-06-12

**Authors:** Lunwu Zeng, Runxia Song

**Affiliations:** Jiangsu Key Laboratory of Intelligent Agricultural Equipment, College of Engineering, Nanjing Agricultural University, Nanjing, 210031 China

## Abstract

Chiral anomaly materials (CAM, e.g., axion insulator, topological insulator and some of Weyl semimetal) are new states of quantum matter. Anomalous Hall effect can occur in CAM, the anomalous Hall effect is closely related to the topological magneto-electric effect, i.e., when an electric field is applied to CAM, not only the electric field is induced, but also the magnetic field, vice versa. According to those properties, we design an electric cloak with quantized CAM and conductor, and a magnetic cloak with quantized CAM and superconductor. Simulation and calculation results show that the electric cloak can cloak applied electric field and induce magnetic field, and the magnetic cloak can cloak applied magnetic field and induce electric field. When applied electric field is generated by a point charge, the monopole can be obtained.

## Introduction

In the past few years, electromagnetic invisibility cloaks have drawn enormous attention. Many achievements of invisibility cloak have been motivated thanks to the pioneering theoretical work^1–4^. Inspired by those theoretical works, varieties of electromagnetic wave cloak^5–11^, acoustic wave cloak^12–14^, matter wave cloak^15^, mass diffusion cloak^16^, heat diffusion cloak^17–20^, magnetic cloak^21–23^, electric cloak^24–26^ have been theoretically designed and experimentally demonstrated. Generally speaking, it is difficult to construct a perfect invisible electromagnetic wave cloak by natural material, for it requires inhomogeneous and anisotropic material, yet a perfect dc electric cloak can be fabricated from natural material. Utilizing resistor network, Yang *et al*. designed anisotropic electric conductance and experimentally demonstrated dc electric cloak^24^, their work shows that the dc electric cloak can guide electric current around the cloaked region smoothly. Utilizing ferromagnetic and superconductor, Gömöry *et al*. experimentally realized a magnetic cloak^22^, which did not require anisotropic metamaterial owing to zero permeability of superconductor. With the similar method, we designed a bi-layer steady current cloak by the use of insulator and conductor^25^, the bi-layer steady current cloak did not require anisotropic metamaterial owing to zero conductivity of insulator either. According to the similarity of the electric conduction equation and heat diffusion equation, the bi-layer heat cloak is designed^19, 20^, the bi-layer heat cloak can shield heat diffusion flux, however, the heat shielding is temporal heat diffusion protection rather than a permanent heat diffusion protection, in the steady state, the cloaked region eventually achieves a constant temperature.

Topological insulator is a new state of quantum matter, the concept of the topological insulator can be defined with topological field theory^27–33^ and topological band theory^34, 35^. Under an applied electric field, a quantized Hall current is induced on the surface of the topological insulator, which in turn generates a magnetic polarization and vice versa, namely, electric field induces magnetic field, and magnetic field induces electric field^27, 28^. Axion insulator is also a new state of quantum matter^36–38^, when an applied electric field is applied on an axion insulator, the electric field can also induce magnetic field, vice versa.

Three type fermions play a fundamental role in our understanding of nature^39^: Dirac, Weyl and Majorana fermions. Dirac fermions have been observed for decades in high-energy physics, Weyl and Majorana fermions have not been observed in high-energy physics, but, Dirac^40–42^, Weyl^38, 39, 43–47^ and Majorana^48–52^ fermions have been discovered in condensate matter. Weyl semimetal^38, 39, 43–47^ is a new state of quantum matter, the chiral anomaly of Weyl semimetal^53–60^ leads to the result that an applied electric field induces magnetic polarization and an applied magnetic field induces electric polarization. Chiral anomaly^27, 37, 38^ would emerge in all topological insulator and axion insulator, some of Weyl semimetal can generate chiral anomaly^53–60^ too, the chiral anomaly^54, 55^ is closely related to the topological magneto-electric response, utilizing the property of topological magneto-electric response, we designed an electric cloak with CAM and conductor bi-layer shell, and a magnetic cloak with CAM and superconductor bi-layer shell. The simulation and calculation results show that the CAM and conductor bi-layer electric cloak can cloak electric field and induce magnetic field; and the CAM and superconductor bi-layer magnetic cloak can cloak magnetic field and induce electric field.

## Results

### Cloaking magnetic field and inducing electric field

The electromagnetic response of three dimensional insulator is described by the Maxwell action *S*_0_ = (8/*π*)∫d^3^ *xdt*(*εE* ^2^ − *B* ^2^/*μ*), together with a topological *θ* term ${S}_{\theta }=(\theta /2\pi )(\alpha /2\pi )\int {{\rm{d}}}^{{\rm{3}}}xdt\overrightarrow{E}\cdot \overrightarrow{B}$ ^27, 28, 60^, here $\overrightarrow{E}$ and $\overrightarrow{B}$ are the electric field and magnetic flux density, respectively, *ε* and *μ* are material-dependent permittivity and permeability, respectively, *α* = *e* ^2^/$\hslash$ *c* is the fine-structure constant, *e* is the electron charge, $\hslash$ is Plank’s constant, *c* is the speed of light, and *θ* is the dimensionless parameter describing the insulator, which refers to the axion field in high-energy physics^60^. *θ* is also called topological magneto-electric polarizability^32, 33^. When time reversal symmetry as well as spatial symmetry like inversion are broken, the topological magneto-electric polarizability *θ* is not quantized^37, 38^ and is very small, for example, the magneto-electric polarizability of *Cr*_2_ *O*_3_ and *BiFeO*_3_ is about 10^−3^ and 10^−4^, respectively^38^. How to improve magneto-electric polarizability? The key point is to quantize the magneto-electric polarizability. When time reversal invariant, the magneto-electric polarizability *θ* is quantized, i.e., *θ* = (2n + 1)*π* (*n* is integral), in this work, we take *θ* = *π* ^37, 38, 53^, i.e., the topological magneto-electric effect was quantized.

Because the electric field induces magnetic polarization and the magnetic field induces electric polarization, the constitute relations of CAM can be written as^31^, (SI unit) $\overrightarrow{D}=\varepsilon {\varepsilon }_{0}\overrightarrow{E}-{({\varepsilon }_{0}/{\mu }_{0})}^{1/2}(\alpha \theta /\pi )\overrightarrow{B}$ and $\overrightarrow{H}=\overrightarrow{B}/\mu {\mu }_{0}+{({\varepsilon }_{0}/{\mu }_{0})}^{1/2}(\alpha \theta /\pi )\overrightarrow{E}$, the constitute relations can be also written as^32^

1 $$\overrightarrow{D}={\varepsilon }_{0}(\varepsilon +\mu \frac{{\alpha }^{2}{\theta }^{2}}{{\pi }^{2}})\overrightarrow{E}-\mu \sqrt{{\varepsilon }_{0}{\mu }_{0}}\frac{\alpha \theta }{\pi }\overrightarrow{H}$$

2 $$\overrightarrow{B}=\mu {\mu }_{0}\overrightarrow{H}-\mu \sqrt{{\varepsilon }_{0}{\mu }_{0}}\frac{\alpha \theta }{\pi }\overrightarrow{E}$$

where *ε*_0_ = 8.85 × 10^−12^ *F*/*m* and *μ*_0_ = 4*π* × 10^−7^ *H*/*m* are the permittivity and permeability of the free space, respectively.

**Figure 1:**
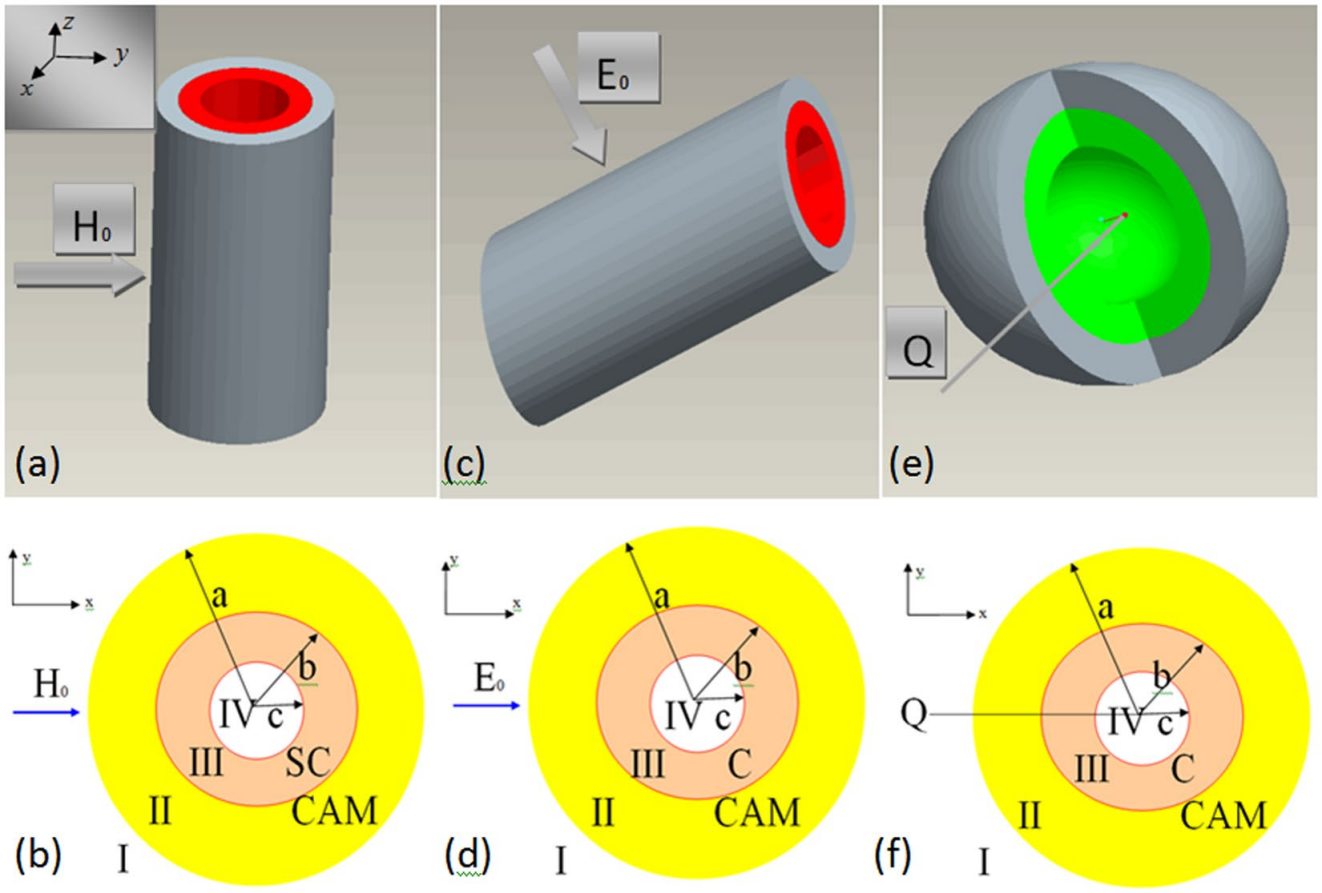
The bi-layer cylindrical and spherical cloak. (**a**) The 3D CAM and superconductor bi-layer cylindrical magnetic cloak. (**b**) The cross section (z = 0, axial) in (**a**), the “SC” stands for superconductor, a uniform magnetic field $\mathop{{H}_{{\rm{0}}}}\limits^{\longrightarrow}$ is applied on the cylindrical magnetic cloak. (**c**) The 3D CAM and conductor bi-layer cylindrical electric cloak. (**d**) The cross section (z = 0, axial) in (**c**), the “C” stands for conductor, a uniform magnetic field $\mathop{{E}_{{\rm{0}}}}\limits^{\longrightarrow}$ is applied on the cylindrical electric cloak. (**e**) The 3D CAM and conductor bi-layer spherical electric cloak. (**f**) The cross section (z = 0) in (**e**), a point electric charge *Q* is in front of the spherical electric cloak.

Figure 1a (3D model) and Fig. 1b (cross section *z* = 0) show the bi-layer cylindrical magnetic cloak, the outer layer is CAM, and the inner layer is superconductor, the permittivity and permeability of the CAM are *ε*_2_ and *μ*_2_, respectively, the permittivity and permeability of the superconductor are *ε*_3_ and *μ*_3_, respectively, the outer radius and inner radius of the CAM are *a* and *b*, respectively, the outer radius and inner radius of the superconductor are *b* and *c*, respectively. The permittivity and permeability of the surroundings are *ε*_1_ and *μ*_1_, respectively, the permittivity and permeability of the cloaked region are *ε*_4_ and *μ*_4_, respectively. When a uniform magnetic field $\mathop{{H}_{0}}\limits^{\longrightarrow}$ is applied to the cloak, the magnetic scalar potential in the cylindrical coordinate system (*r*, *θ*, *z*) in the four regions can be written as^22^ ${\varphi }_{m1}=(-{H}_{0}r+A/r)\cos \,\theta$, ${\varphi }_{m2}=(Br+C/r)\cos \,\theta$, ${\varphi }_{m3}=(Dr+E/r)\cos \,\theta$, ${\varphi }_{m4}=Fr\,\cos \,\theta$, where *A*, *B*, *C*, *D*, *E* and *F* are unknown coefficients. The relation between the magnetic field and the magnetic scalar potential is $\overrightarrow{H}=-\nabla {\varphi }_{m}$, where $\overrightarrow{H}$ is the magnetic field, *φ*_*m*_ is the magnetic scalar potential, $\nabla =\partial /\partial r\overrightarrow{{e}_{r}}+(1/r)\partial /\partial \theta \overrightarrow{{e}_{\theta }}+\partial /\partial z\overrightarrow{{e}_{z}}$, $\overrightarrow{{e}_{r}}$ is normal unit vector, $\overrightarrow{{e}_{\theta }}$ is tangential unit vector, the magnetic field in the four regions can be obtained (See Supplementary Information). Due to the magneto-electric response of the CAM, the electric potential is induced in the four regions can be written as ${\varphi }_{e1}=({A}_{0}r+{A}_{1}/r)\cos \,\theta$, ${\varphi }_{e2}=({B}_{1}r+{C}_{1}/r)\cos \,\theta$, ${\varphi }_{e3}=({D}_{1}r+{E}_{1}/r)\cos \,\theta$, ${\varphi }_{e4}={F}_{1}r\,\cos \,\theta$, where *A*_0_, A_1_, *B*_1_, *C*_1_, *D*_1_, *E*_1_ and *F*_1_ are unknown coefficients. Because of no physics electric field in region I, so *A*_0_ = 0. The relation between the electric field and the electric potential is $\overrightarrow{E}=-\nabla {\varphi }_{e}$, the electric field in the four regions can be obtained (See Supplementary Information). When *A* = 0, the magnetic field is still *H*_0_ in region I, when *F* = 0, the magnetic field vanishes in region IV, namely, the device is a magnetic cloak. When A_1_, *B*_1_, *C*_1_, *D*_1_, *E*_1_ and *F*_1_ are non-zero, the electric field is induced by the applied magnetic field. That is to say, we can cloak the magnetic field and induce electric field. The calculation results show that it is impossible to obtain a cloak when the inner layer and the outer layer are both the CAM, however, when the outer layer is CAM, the inner layer is superconductor, the bi-layer cylinder can cloak applied magnetic field and induce electric field.

**Figure 2:**
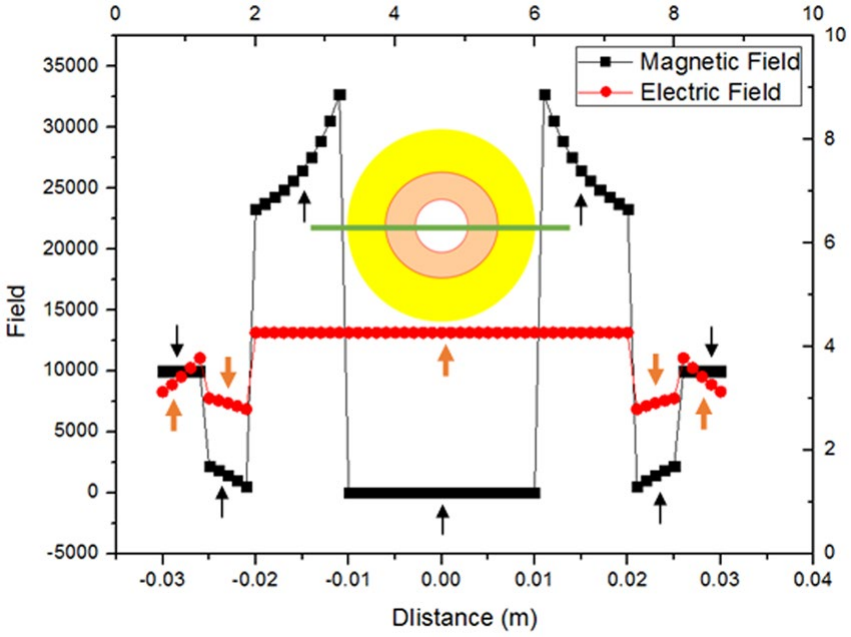
The relation between the permeability *μ*_2_ and the radii ratios *a*/*b*. The different radii ratios and correspondingly permeability can obtain the same cloaked effect.

Supposed that the inner layer is a superconductor, namely, the magneto-electric polarizability *θ*_3_ = 0, the permeability of the superconductor *μ*_3_ = 0, we take the permittivity of the superconductor *ε*_3_ = 1. The outer layer is CAM, the quantized magneto-electric polarizability of the CAM is *θ*_2_ = *π*, the permittivity of the CAM *ε*_2_ = 2, the fine-structure constant *α* = 1/137, the other parameters are *ε*_1_ = 1, *ε*_4_ = 1, *μ*_1_ = 1, *μ*_4_ = 1, setting *A* = 0, *F* = 0, we can obtain the conditions of the cloak, the relations between the size parameters and material parameters are

3 $${\mu }_{2}=\frac{({a}^{2}+{b}^{2})[{a}^{2}{({\varepsilon }_{2}+1)}^{2}-{b}^{2}{({\varepsilon }_{2}-1)}^{2}]}{X+Y+Z},$$

where, *X* = 4(*αθ*_2_/*π*)^2^ *a* ^2^ *b* ^2^, $Y=-{(\alpha {\theta }_{2}/\pi )}^{2}({a}^{2}+{b}^{2})[{a}^{2}({\varepsilon }_{2}+1)-{b}^{2}({\varepsilon }_{2}-1)]$, *Z* = (*a* ^2^ − *b* ^2^)[*a* ^2^(*ε*_2_ + 1)^2^ − *b* ^2^(*ε*_2_ − 1)^2^]. Figure 2 shows the relations between the radii ratios *a*/*b* and the permeability *μ*_2_, for example, if *a*/*b* = 5, *μ*_2_ = 1.083, then *A* = 0, *F* = 0; if *a*/*b* = 1.25, *μ*_2_ = 4.556, then *A* = 0, *F* = 0.

**Figure 3:**
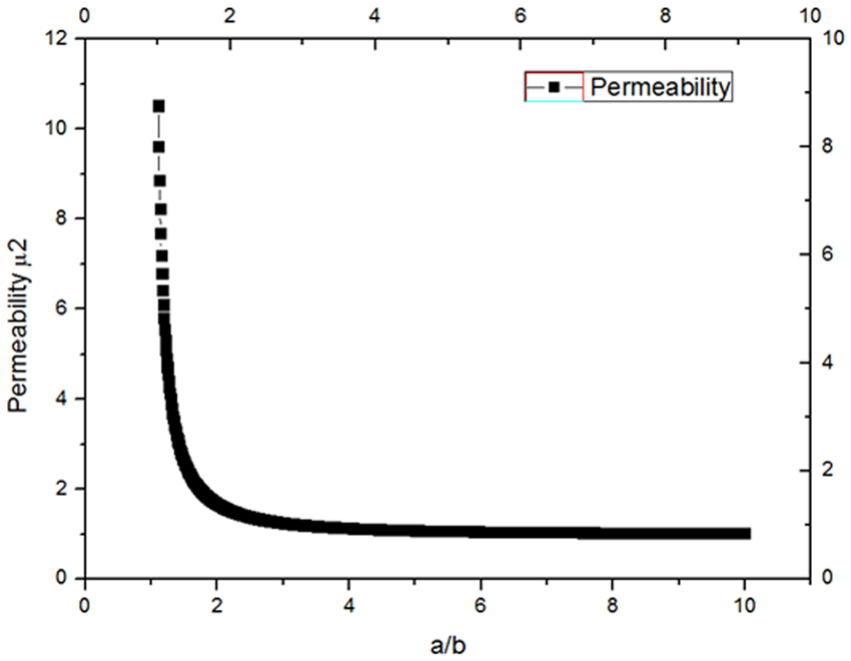
The distributions of the magnetic field and induced electric field in the center line (y = 0, z = 0, green line in insert). The seven black segments (indicated by black arrows) are the distributions of the magnetic field and the five red segments (indicated by red arrows) are the distributions of the induced electric field. The magnetic field and induced electric field are symmetry about x = 0, the electric and the magnetic field are not continuous on the boundaries x = ±0.025 m, x = ±0.020 m, x = ±0.010 m.

Figure 3 shows the distributions of the magnetic field and the electric field in the center line (y = 0, namely, the green line in insert) of the cross section (z = 0), the black lines stand for the magnetic field in the center line (y = 0, z = 0) of the cloak, and the red lines stand for the electric field in the center line (y = 0, z = 0) of the cloak, the parameters are *a* = 0.025 *m*, *b* = 0.020 *m*, *c* = 0.010 *m*, H_0_ = 10000 A/m, *μ*_2_ = 4.556, other parameters are the same in the Fig. 2. When *r *> 0.025 m, the magnetic field H_1_ = H_0_ = 10000 A/m; the induced electric field E_1_ varies with size parameters a and b, and has nothing to do with the size parameter c; the induced electric field E_1_ varies with material parameter *ε*, less influenced by material parameter *μ*. When 0.020 m < *r* < 0.025 m, the magnetic field H_2_ and the induced electric field E_2_ both vary with size parameters a and b, and have nothing to do with the size parameter c; the magnetic field H_2_ varies with material parameter *μ*, less influenced by material parameter *ε*; the induced electric field E_2_ varies with material parameters *ε*, less influenced by material parameter *μ*. When 0.010 m < *r* < 0.020 m, the magnetic field H_3_ varies with size parameters a, b, c and material parameters *μ*, less influenced by material parameter *ε*; the induced electric field E_3_ = 13166 V/*m* is uniform. Noting that the magnetic flux density is zero in this region, this is because the superconductor (*μ*_3_ = 0) repulse magnetic flux density. When *r* < 0.010 m, the magnetic field H_4_ = 0, the induced electric field E_4_ = 13166 V/*m* is uniform, namely, the CAM and superconductor bi-layer cylinder cloaked applied magnetic field and induced electric field. The induced electric field E_3_ = E_4_ = 13166 V/*m* is uniform in the superconductor (0.010 *m* < *r* < 0.02 m), this is because we chose the permittivity of the superconductor *ε*_3_ = 1, when *ε*_3_ ≠ 1, the induced electric field E_3_ is not uniform in the superconductor. When *θ*_2_ = 0, *θ*_3_ = 0, *μ*_3_ = 0, the CAM and superconductor bi-layer magnetic cloak is simplified as a conventional magnetic cloak^22, 23^, utilizing ferromagnetic and superconductor bi-layer material, Gömöry *et al*. experimentally demonstrated the magnetic cloak^22^. The cloak is also influenced by magneto-electric polarizability, if the magneto-electric polarizability is quantized, then *θ* = *π*; if the magneto-electric polarizability is not quantized, then *θ* is very small^38^, in this case, the electric field induced by magnetic field can be ignored, and the magnetic field induced by electric field can be ignored too.

### Cloaking electric field and inducing magnetic field

Figure 1c (3D model) and Fig. 1d (cross section *z* = 0) show the bi-layer cylindrical electric cloak, the outer layer is CAM, and the inner layer is conductor. When a uniform electric field $\mathop{{E}_{0}}\limits^{\longrightarrow}$ is applied, the electric potential in the cylindrical coordinate system (*r*, *θ*, *z*) in the four regions can be written as^22^ ${\varphi }_{e1}=(-{E}_{0}r+A/r)\cos \,\theta$, ${\varphi }_{e2}=(Br+C/r)\cos \,\theta$, ${\varphi }_{e3}=(Dr+E/r)\cos \,\theta$, ${\varphi }_{e4}=Fr\,\cos \,\theta$, where *A*, *B*, *C*, *D*, *E* and *F* are unknown coefficients. The relation between the electric field and the electric potential is $\overrightarrow{E}=-\nabla {\varphi }_{e}$, the electric field in the four regions can be obtained (See Supplementary Information). Due to the topological magneto-electric response of the CAM, namely, the electric field induces magnetic field, the magnetic scalar potential in the four regions is induced ${\varphi }_{m1}=({A}_{0}r+{A}_{1}/r)\cos \,\theta$, ${\varphi }_{m2}=({B}_{1}r+{C}_{1}/r)\cos \,\theta$, ${\varphi }_{m3}=({D}_{1}r+{E}_{1}/r)\cos \,\theta$, ${\varphi }_{m4}={F}_{1}r\,\cos \,\theta$, where *A*_0_, A_1_, *B*_1_, *C*_1_, *D*_1_, *E*_1_ and, *F*_1_ are unknown coefficients. The relation between the magnetic field and the magnetic scalar potential is $\overrightarrow{H}=-\nabla {\varphi }_{m}$, the magnetic field in the four regions can also be obtained (See Supplementary Information). Because of no physics magnetic field in region I, so *A*_0_ = 0. When *A* = 0, the electric field is still *E*_0_ in region I; when *F* = 0, the electric field vanishes in region IV, namely, the device is an electric cloak. When A_1_, *B*_1_, *C*_1_, *D*_1_, *E*_1_, and *F*_1_ are non-zero, the magnetic fields are induced by the applied electric field. That is to say, we can cloak the electric field and induce magnetic field. The calculation results show that it is impossible to obtain a cloak when the inner layer and the outer layer are both the CAM, however, when the outer layer is CAM, the inner layer is conductor, the bi-layer cylinder can cloak electric field and induce magnetic field. Supposed that the inner layer is a conductor, namely, the magneto-electric polarizability of the conductor is *θ*_3_ = 0, the permittivity of conductor is *ε*_3_ → ∞, setting *A* = 0, *F* = 0, we can obtain the conditions of the electric cloak.

**Figure 4:**
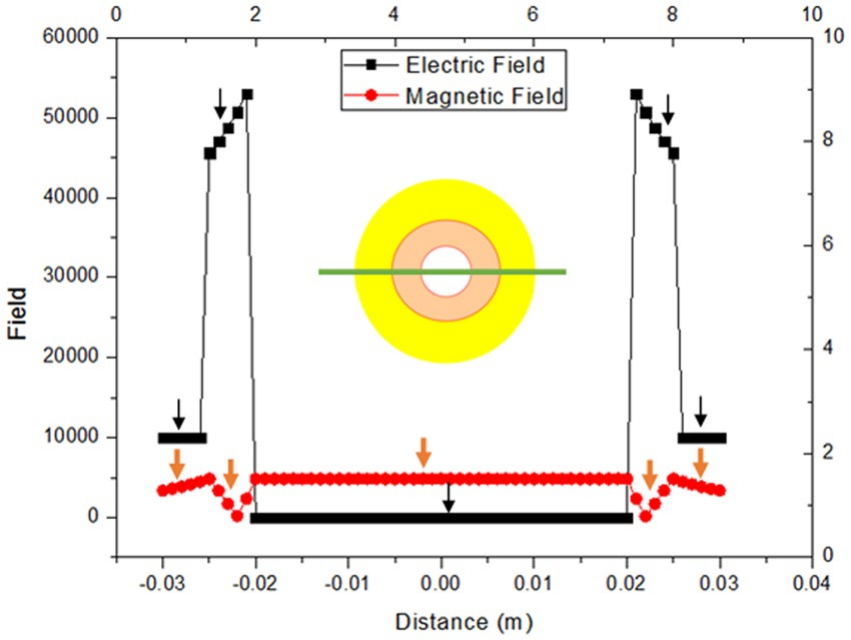
The distributions of the electric field and induced magnetic field in the center line (y = 0, z = 0, green line in insert). The five black segments (indicated by black arrows) are the distributions of the electric field and the five red segments (indicated by red arrows) are the distributions of the induced magnetic field. The electric field and induced magnetic field are symmetry about x = 0, the electric field and the magnetic field are not continuous on the boundaries x = ±0.025 m, x = ±0.020 m, x = ±0.010 m. The magnetic fields are 50000 times actual magnetic field.

Figure 4 shows the distributions of the electric field and the magnetic field in the center line (y = 0, namely, the green line in insert) of the cross section (z = 0), the black lines stand for the electric field in the center line (y = 0, z = 0) of the cloak, and the red lines stand for the magnetic field in the center line (y = 0, z = 0) of the cloak, the applied electric field E_0_ = 10000 V/m, the permittivity of the surrounding and the conductor is *ε*_1_ = 9.11 and *ε*_3_ → ∞, respectively, the magneto-electric polarizability of the conductor is *θ*_3_ = 0, the other parameters are the same in the Fig. 3. When *r* > 0.025 m, the electric field E_1_ = E_0_ = 10000 V/m; the induced magnetic field H_1_ varies with size parameters a and b, and has nothing to do with the size parameter c; the induced magnetic field H_1_ varies with material parameter *μ*, less influenced by material parameter *ε*. When 0.020 m < *r* < 0.025 m, the electric field E_2_ and the induced magnetic field H_2_ both vary with size parameters a and b, and have nothing to do with the size parameter c; the electric field E_2_ varies with material parameters *ε*, less influenced by material parameter *μ*; the induced magnetic field H_2_ varies with material parameters *μ*, less influenced by material parameter *ε*. When 0.010 m < *r* < 0.020 m, the electric field E_3_ = 0, this is because the electric field is zero in conductor, yet the induced magnetic field H_3_ = 0.0968 A/*m*. When *r* < 0.010 m, the electric field E_4_ = 0, the induced magnetic field H_4_ = 0.0968 A/*m* is uniform. Namely, the CAM and conductor bi-layer can cloak applied electric field and induce magnetic field, the induced magnetic field is very weak. Noting that H_3_ = H_4_ = 0.0968 A/*m*, this is because we chose *μ*_3_ = *μ*_4_ = 1, if *μ*_3_ ≠ *μ*_4_, then H_3_ ≠ H_4_. If *θ*_2_ = 0, *θ*_3_ = 0, our cloak is simplified as a conventional electric cloak, utilizing dielectric and conductor, Lan *et al*. designed bi-layer electrostatic cloak, and experimentally demonstrated bi-layer electrostatic cloak^61^. When *ε*_3_ = 0 (the permittivity of the dual superconductor^62^ is zero), the CAM and dual superconductor bi-layer electric cloak can be designed. Noting that *ε*_1_ = 9.11 is very large, we can decrease *ε*_1_ by adjusting size parameters *a* and *b*.

### Cloaking electric field of point electric charge and inducing monopole

Figure 1e (3D model) and Fig. 1f (cross section *z* = 0) show the bi-layer spherical cloak, the outer layer is CAM, and the inner layer is conductor, *Q* is a point electric charge. The electric potential in the four regions is (See Supplementary Information) ${\varphi }_{e1}=\sum (-(Q/4\pi {\varepsilon }_{0})({r}^{n}/{L}^{n+1})+{A}_{n}/{r}^{n+1}){P}_{n}(\cos \,\theta )$, ${\varphi }_{e2}=\sum ({B}_{n}{r}^{n}+{C}_{n}/{r}^{n+1}){P}_{n}(\cos \,\theta )$, ${\varphi }_{e3}=\sum ({D}_{n}{r}^{n}+{E}_{n}/{r}^{n+1})$ ${P}_{n}(\cos \,\theta )$, ${\varphi }_{e4}=\sum ({F}_{n}{r}^{n}){P}_{n}(\cos \,\theta )$, where *A*_*n*_, *B*_*n*_, *C*_*n*_, *D*_*n*_, *E*_*n*_ and *F*_*n*_ are unknown coefficients, it should be pointed out that *φ*_*e*2_, *φ*_*e*3_ and *φ*_*e*4_ are the electric potential superposition of the point electric charge and spherical cloak. The relation between the electric field and electric potential is $\overrightarrow{E}=-\nabla {\varphi }_{e}$, $\nabla =\partial /\partial r\overrightarrow{{e}_{r}}+(1/r)\partial /\partial \theta \overrightarrow{{e}_{\theta }}+(1/r\,\sin \,\theta )\partial /\partial \varphi \overrightarrow{{e}_{\varphi }}$, the electric field in the four regions can be obtained (See Supplementary Information). Due to the magneto-electric response of the CAM, the magnetic scalar potential is induced in the four regions, ${\varphi }_{m1}=\sum ({A}^{{\rm{01}}}{r}^{n}+{A}_{n}^{1}/{r}^{n+1}){P}_{n}(\cos \,\theta )$, ${\varphi }_{m2}=\sum ({B}_{n}^{1}{r}^{n}+{C}_{n}^{1}/{r}^{n+1}){P}_{n}(\cos \,\theta )$, ${\varphi }_{m3}=\sum ({D}_{n}^{1}{r}^{n}+{E}_{n}^{1}/{r}^{n+1}){P}_{n}(\cos \,\theta )$, ${\varphi }_{m4}=\sum {F}_{n}{r}^{n}{P}_{n}(\cos \,\theta )$, where *A* ^01^, ${{\rm{A}}}_{{\rm{n}}}^{{\rm{1}}}$, ${{\rm{B}}}_{{\rm{n}}}^{{\rm{1}}}$, ${{\rm{C}}}_{{\rm{n}}}^{{\rm{1}}}$, ${{\rm{D}}}_{{\rm{n}}}^{{\rm{1}}}$, ${{\rm{E}}}_{{\rm{n}}}^{{\rm{1}}}$ and ${{\rm{F}}}_{{\rm{n}}}^{{\rm{1}}}$ are unknown coefficients. Because of no physics magnetic field in region I, so *A* ^01^ = 0. We can obtain the magnetic field in the four regions (See Supplementary Information). When A_n_ = 0, only the electric field of the point electric charge is in region I; when *F*_*n*_ = 0, the electric field vanishes in region IV, namely, the device is an electric cloak. When ${{\rm{A}}}_{{\rm{n}}}^{{\rm{1}}}$, ${{\rm{B}}}_{{\rm{n}}}^{{\rm{1}}}$, ${{\rm{C}}}_{{\rm{n}}}^{{\rm{1}}}$, ${{\rm{D}}}_{{\rm{n}}}^{{\rm{1}}}$, ${{\rm{E}}}_{{\rm{n}}}^{{\rm{1}}}$ and ${{\rm{F}}}_{{\rm{n}}}^{{\rm{1}}}$ are non-zero, the magnetic field is induced by the point electric charge. That is to say, we can cloak the electric field and induce magnetic field. Supposed that the inner layer is a conductor, setting *A*_*n*_ = 0, *F*_*n*_ = 0, solving the boundary conditions, we can obtain ${{\rm{A}}}_{{\rm{n}}}^{{\rm{1}}}$, ${{\rm{B}}}_{{\rm{n}}}^{{\rm{1}}}$, ${{\rm{C}}}_{{\rm{n}}}^{{\rm{1}}}$, ${{\rm{D}}}_{{\rm{n}}}^{{\rm{1}}}$, ${{\rm{E}}}_{{\rm{n}}}^{{\rm{1}}}$ and ${{\rm{F}}}_{{\rm{n}}}^{{\rm{1}}}$, according to magnetic charge formulation^63^, the interesting thing take places, namely, many monopoles are induced in the four regions. For example, neglecting infinitesimal of the higher order (See Supplementary Information), we obtain coefficient ${A}_{n}^{1}=-(\alpha {\varepsilon }_{1}/{\varepsilon }_{2}){({\varepsilon }_{0}/{\mu }_{0})}^{1/2}(Q/4\pi {\varepsilon }_{0})n/(2n+1){a}^{2n+1}/{L}^{n+1}$, and the magnetic scalar potential in region I,

4 $${\varphi }_{m{\rm{1}}}=-\frac{\alpha {\varepsilon }_{1}}{{\varepsilon }_{2}}\sqrt{\frac{{\varepsilon }_{0}}{{\mu }_{0}}}\frac{Q}{4\pi {\varepsilon }_{0}}\sum (\frac{n}{2n+1}\frac{{a}^{2n+1}}{{L}^{n+1}{r}^{n+1}}){P}_{n}(\cos \,\theta )$$

That is to say, the monopole is induced by a point electric charge, the monopole charge may be positive or negative, the positive or negative is decided by angle *θ*. Expanding Eq. (4), we can obtain a monopole in the inversion point (See Supplementary Information).

**Figure 5:**
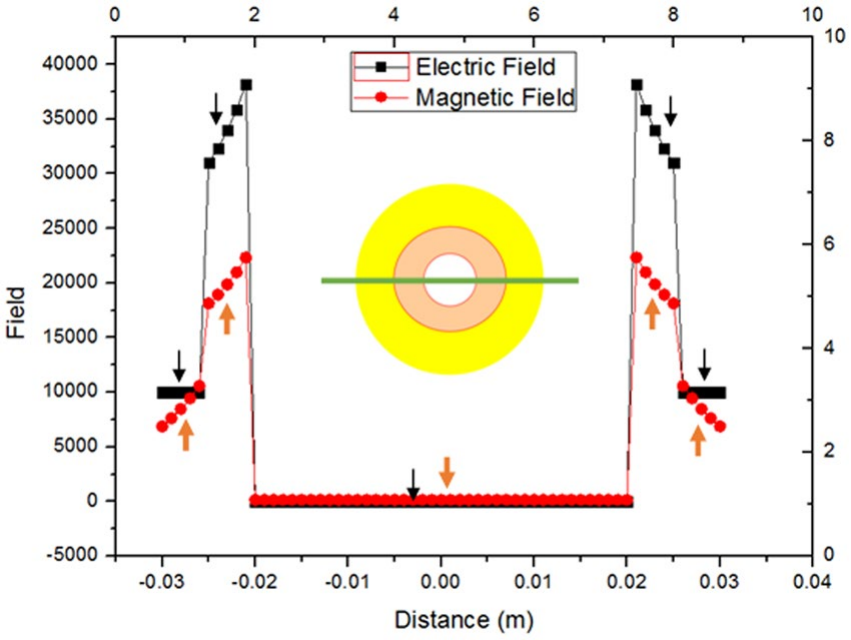
The distributions of the electric field and induced magnetic field in the center line (y = 0, z = 0, green line in insert). The five black segments (indicated by black arrows) are the distributions of the electric field and the five red segments (indicated by red arrows) are the distributions of the induced magnetic field. The electric and the induced magnetic field are not continuous on the boundaries x = ±0.025 m, x = ±0.020 m, x = ±0.010 m. The magnetic fields are 30000 times actual magnetic field.

Figure 5 shows the distributions of the electric field and the magnetic field in the center line (y = 0, namely, the green line in insert) of the cross section (z = 0), the black lines stand for the electric field in the center line (y = 0, z = 0) of the cloak, and the red lines stand for the induced magnetic field in the center line (y = 0, z = 0) of the cloak, the parameters are *n* = 1, Q = 1.0 × 10^−7^ *C*, L = 0.30 m, *ε*_1_ = 8.295, the other parameters are the same as in Fig. 4. When *r* > 0.025 m, the electric field is the field of the point electric charge, the induced magnetic field varies with space. When 0.020 m < *r* < 0.025 m, the electric field and the induced magnetic field both vary with space. When 0.010 m < *r* < 0.020 m, the electric field is equal to zero, this is because the electric field is zero in conductor, the magnetic field H_3_ = 0.0048 A/*m* is uniform, noting that the magnetic field is a uniform field in this region, this is because we chose the permeability of the CAM *μ*_3_ = 1, when *μ*_3_ ≠ 1, the magnetic field is not uniform. When *r* < 0.010 m, the electric field is equal to zero, the magnetic field H_4_ = 0.0048 A/*m* is uniform. Namely, the CAM and conductor spherical bi-layer cloaked applied electric field and induced magnetic field, the induced magnetic field is rather weak.

Utilizing quantized Faraday and Kerr rotation effect of topological insulator, Wu *et al*. measured fine-structure constant^64^. By measuring electric field or magnetic field of our cloaks, we can also measure the fine-structure constant, Plank’s constant, topological magneto-electric polarizability and electron charge. It offers a new way for designing various magneto-electric material cloaks such as carpet cloak^62^, phase shift cloak^65^. The topological insulator^27^ as a magneto-electric material can cloak electric field and induce magnetic field (or monopole), or cloak magnetic field and induce electric field, yet most of topological insulators (for example *B*i_2_ *Se*_3_) involve p-electron orbit^37, 38^, whose Coulomb interaction is weak and cannot support magnetism, the control ability to magnetic field is weak. The axion insulator^37, 38^ as a magneto-electric material can also cloak electric field and induce magnetic field (or monopole), or cloak magnetic field and induce electric field. The axion insulator can be obtained by the Coulomb correlation and exotic electronic properties, for example, when Coulomb correlation U < 0.4 eV, compound *CaOs*_2_ *O*_4_ is a metal; when 0.4 eV < U < 0.9 eV, compound *CaOs*_2_ *O*_4_ is an axion insulator; when 0.9 eV < U < 1.4 eV, compound *CaOs*_2_ *O*_4_ is a Weyl semimetal metal; when U > 1.4 eV, compound *CaOs*_2_ *O*_4_ is a Mott insulator^38^.

## Conclusion

Utilizing CAM and superconductor (conductor), we designed bi-layer cloak. According to the electromagnetic field theory, we obtain the magnetic scalar potential and electric potential. We demonstrated that the CAM and superconductor bi-layer can cloak applied magnetic field and induce electric field, and the CAM and conductor bi-layer can cloak applied electric field and induce magnetic field. Those cloaks can be experimentally demonstrated and be applied to electric and magnetic field switch, namely, magnetic field controls electric field or electric field controls magnetic field. By measuring electric field and magnetic field, we can measure the fine-structure constant or topological magneto-electric polarizability.

## Methods

In the conventional material, the electric field induces electric polarization; the magnetic field induces magnetic polarization. In the chiral anomaly materials, e.g., axion insulator, topological insulator and some of Weyl semimetal, the anomalous Hall effect emerge^57, 66^, the anomalous Hall effect is closely related to the topological magneto-electric effect, i.e., when an electric field is applied to CAM, both the electric and magnetic field are induced. Utilizing those properties, the constitute relations of the CAM are obtained $\overrightarrow{D}={\varepsilon }_{0}(\varepsilon +\mu {\alpha }^{2}{\theta }^{2}/{\pi }^{2})\overrightarrow{E}-\mu \alpha \theta \sqrt{{\varepsilon }_{0}{\mu }_{0}}/\pi \overrightarrow{H}$, $\overrightarrow{B}=\mu {\mu }_{0}\overrightarrow{H}-\mu \alpha \theta \sqrt{{\varepsilon }_{0}{\mu }_{0}}/\pi \overrightarrow{E}$. Considering the symmetry, in the cylindrical coordinate system (*r*, *θ*, *z*), the solution of the Laplace equation is $\varphi =\sum ({M}_{n}{r}^{n}+{N}_{n}/{r}^{n}){P}_{n}(\cos \,\theta )$; in the spherical coordinate system (*r*, *θ*, *φ*), the solution of the Laplace equation is $\varphi =\sum ({M}_{n}{r}^{n}+{N}_{n}/{r}^{n+{\rm{1}}}){P}_{n}(\cos \,\theta )$. According to relation between the electric field and the electric potential $\overrightarrow{E}=-\nabla {\varphi }_{e}$, and the magnetic field and the magnetic scalar potential $\overrightarrow{H}=-\nabla {\varphi }_{m}$, we obtain magnetic field and magnetic scalar potential in different regions, according to the constitute relations of the CAM, and the boundary conditions of the electric field and magnetic field, we obtain the conditions of the cloak. Utilizing the Legendre mother function, we expand the electric potential of point electric charge (See Supplementary Information).

## Electronic supplementary material

Supplementary Information for “Quantized chiral anomaly materials cloak”
