# Supplementary material for: Quantized chiral anomaly materials cloak
Source: Sci Rep. 2017 Jun 12;7:3253. doi: 10.1038/s41598-017-03587-y (PMC5468267; doi:10.1038/s41598-017-03587-y)
Supplement: Supplementary file 1 — Supplementary Information for “Quantized chiral anomaly materials cloak” [file 41598_2017_3587_MOESM1_ESM.doc]

**Supplementary Information for “Quantized** **chiral anomaly materials cloak”**

**Lunwu Zeng, and Runxia Song**

***Jiangsu Key Laboratory of Intelligent Agricultural Equipment, College of Engineering, Nanjing Agricultural University, Nanjing 210031, China***

1. **Cloaking magnetic field and inducing electric field****.** The constitute relations of the chiral anomaly materials (CAM) are

$\overrightarrow{D}=\varepsilon_{0}(\varepsilon+\mu\frac{\alpha^{2}\theta^{2}}{\pi^{2}})\overrightarrow{E}-\mu\sqrt{\varepsilon_{0}\mu_{0}}\frac{\alpha\theta}{\pi}\overrightarrow{H}$ (A1)

$\overrightarrow{B}=\mu\mu_{0}\overrightarrow{H}-\mu\sqrt{\varepsilon_{0}\mu_{0}}\frac{\alpha\theta}{\pi}\overrightarrow{E}$ (A2)

I

II

III

SC

IV

CAM

Fig. A1. The CAM and superconductor (SC) bi-layer cylindrical magnetic cloak.

See Fig. A1, the bi-layer cloak is made of CAM and superconductor. When a uniform magnetic field $\overrightarrow{H_{0}}$ is applied, the magnetic scalar potential in the cylindrical coordinate system $(r,\theta,z)$ in the four regions can be written as

$\phi_{m1}=(-H_{0}r+\frac{A}{r})\cos\theta$ (A3)

$\phi_{m2}=(Br+\frac{C}{r})\cos\theta$ (A4)

$\phi_{m3}=(Dr+\frac{E}{r})\cos\theta$ (A5)

$\phi_{m4}=Fr\cos\theta$ (A6)

where $\phi_{m1}$, $\phi_{m2}$, $\phi_{m3}$ and $\phi_{m4}$ are the magnetic scalar potential in the four regions, and $A$, $B$, $C$, $D$, $E$ and $F$ are unknown coefficients. The magnetic field in the four regions can be written as

$\overrightarrow{H}_{1}=-\nabla\phi_{m1}=(H_{0}+\frac{A}{r^{2}})\cos\theta\overrightarrow{e_{r}}+(-H_{0}+\frac{A}{r^{2}})\sin\theta\overrightarrow{e_{\theta}}$ (A7)

$\overrightarrow{H}_{2}=-\nabla\phi_{m2}=(-B+\frac{C}{r^{2}})\cos\theta\overrightarrow{e_{r}}+(B+\frac{C}{r^{2}})\sin\theta\overrightarrow{e_{\theta}}$ (A8)

$\overrightarrow{H}_{3}=-\nabla\phi_{m3}=(-D+\frac{E}{r^{2}})\cos\theta\overrightarrow{e_{r}}+(D+\frac{E}{r^{2}})\sin\theta\overrightarrow{e_{\theta}}$ (A9)

$\overrightarrow{H}_{4}=-\nabla\phi_{m4}=-F\cos\theta\overrightarrow{e_{r}}+F\sin\theta\overrightarrow{e_{\theta}}$ (A10)

Due to the magneto-electric response of the CAM, The electric potential in the four regions is induced

$\phi_{e1}=(A_{0}r+\frac{A_{1}}{r})\cos\theta$ (A11)

$\phi_{e2}=(B_{1}r+\frac{C_{1}}{r})\cos\theta$ (A12)

$\phi_{e3}=(D_{1}r+\frac{E_{1}}{r})\cos\theta$ (A13)

$\phi_{e4}=F_{1}r\cos\theta$ (A14)

where $\phi_{e1}$, $\phi_{e2}$, $\phi_{e3}$ and $\phi_{e4}$ are the electric potential in the four regions, and $A_{0}$, $\text{A}_{1}$, $B_{1}$, $C_{1}$, $D_{1}$, $E_{1}$ and $F_{1}$ are unknown coefficients. Because of no physics electric field in region I, so $A_{0}=0$. The electric field in the four regions can be written as

$\overrightarrow{E}_{1}=-\nabla\phi_{e1}=\frac{A_{1}}{r^{2}}\cos\theta\overrightarrow{e_{r}}+\frac{A_{1}}{r^{2}}\sin\theta\overrightarrow{e_{\theta}}$ (A15)

$\overrightarrow{E}_{2}=-\nabla\phi_{e2}=(-B_{1}+\frac{C_{1}}{r^{2}})\cos\theta\overrightarrow{e_{r}}+(B_{1}+\frac{C_{1}}{r^{2}})\sin\theta\overrightarrow{e_{\theta}}$ (A16)

$\overrightarrow{E}_{3}=-\nabla\phi_{e3}=(-D_{1}+\frac{E_{1}}{r^{2}})\cos\theta\overrightarrow{e_{r}}+(D_{1}+\frac{E_{1}}{r^{2}})\sin\theta\overrightarrow{e_{\theta}}$ (A17)

$\overrightarrow{E}_{4}=-\nabla\phi_{e4}=-F_{1}\cos\theta\overrightarrow{e_{r}}+F_{1}\sin\theta\overrightarrow{e_{\theta}}$ (A18)

The magnetic field is continuous in the tangential direction (or the magnetic scalar potential is continuous),

$-H_{0}+\frac{A}{a^{2}}=B+\frac{C}{a^{2}}$ (A19)

$D+\frac{E}{b^{2}}=B+\frac{C}{b^{2}}$ (A20)

$D+\frac{E}{c^{2}}=F$ (A21)

The electric field is continuous in the tangential direction (or the electric potential is continuous),

$\frac{A_{1}}{a^{2}}=B_{1}+\frac{C_{1}}{a^{2}}$ (A22)

$D_{1}+\frac{E_{1}}{b^{2}}=B_{1}+\frac{C_{1}}{b^{2}}$ (A23)

$D_{1}+\frac{E_{1}}{c^{2}}=F_{1}$ (A24)

The electric displacement is continuous in the normal direction, according to Eq. (A1), we obtain

$\varepsilon_{0}\varepsilon_{1}\frac{A_{1}}{a^{2}}=\varepsilon_{0}(\varepsilon_{2}+\mu_{2}\frac{\alpha^{2}\theta_{2}^{2}}{\pi^{2}})(-B_{1}+\frac{C_{1}}{a^{2}})-\mu_{2}\sqrt{\varepsilon_{0}\mu_{0}}\frac{\alpha\theta_{2}}{\pi}(-B+\frac{C}{a^{2}})$ (A25)

$\varepsilon_{0}(\varepsilon_{2}+\mu_{2}\frac{\alpha^{2}\theta_{2}^{2}}{\pi^{2}})(-B_{1}+\frac{C_{1}}{b^{2}})-\mu_{2}\sqrt{\varepsilon_{0}\mu_{0}}\frac{\alpha\theta_{2}}{\pi}(-B+\frac{C}{b^{2}})$ $=\varepsilon_{0}(\varepsilon_{3}+\mu_{3}\frac{\alpha^{2}\theta_{3}^{2}}{\pi^{2}})(-D_{1}+\frac{E_{1}}{b^{2}})-\mu_{3}\sqrt{\varepsilon_{0}\mu_{0}}\frac{\alpha\theta_{3}}{\pi}(-D+\frac{E}{b^{2}})$ (A26)

$\varepsilon_{0}(\varepsilon_{3}+\mu_{3}\frac{\alpha^{2}\theta_{3}^{2}}{\pi^{2}})(-D_{1}+\frac{E_{1}}{c^{2}})-\mu_{3}\sqrt{\varepsilon_{0}\mu_{0}}\frac{\alpha\theta_{3}}{\pi}(-D+\frac{E}{c^{2}})=-\varepsilon_{0}\varepsilon_{4}F_{1}$ (A27)

The magnetic flux density is continuous in the normal direction, according to Eq. (A2), we obtain

$\mu_{0}\mu_{1}(H_{0}+\frac{A}{a^{2}})=\mu_{0}\mu_{2}(-B+\frac{C}{a^{2}})-\mu_{2}\sqrt{\varepsilon_{0}\mu_{0}}\frac{\alpha\theta_{2}}{\pi}(-B_{1}+\frac{C_{1}}{a^{2}})$ (A28)

$\mu_{0}\mu_{2}(-B+\frac{C}{b^{2}})-\mu_{2}\sqrt{\varepsilon_{0}\mu_{0}}\frac{\alpha\theta_{2}}{\pi}(-B_{1}+\frac{C_{1}}{b^{2}})$ $=\mu_{0}\mu_{3}(-D+\frac{E}{b^{2}})-\mu_{3}\sqrt{\varepsilon_{0}\mu_{0}}\frac{\alpha\theta_{3}}{\pi}(-D_{1}+\frac{E_{1}}{b^{2}})$ (A29)

$\mu_{0}\mu_{3}(-D+\frac{E}{c^{2}})-\mu_{3}\sqrt{\varepsilon_{0}\mu_{0}}\frac{\alpha\theta_{3}}{\pi}(-D_{1}+\frac{E_{1}}{c^{2}})=-\mu_{0}\mu_{4}F$ (A30)

When $A=0$, the magnetic field is still $H_{0}$ in region I, when $F=0$, the magnetic field vanishes in region IV, namely, when $\text{A}_{1}$, $B_{1}$, $C_{1}$, $D_{1}$, $E_{1}$ and $F_{1}$ are non-zero, the electric field is induced by the applied magnetic field.

Setting $A=0$, $F=0$, solving Eq. (A19) - Eq. (A30), we can obtain the conditions of the cloak, the relations between the size parameters and material parameters are

$\mu_{2}=\frac{(a^{2}+b^{2})[a^{2}(\varepsilon_{2}+1)^{2}-b^{2}(\varepsilon_{2}-1)^{2}]}{X+Y+Z}$, (A31)

where, $X=4\text{（}\alpha\theta_{2}/\pi\text{）}^{2}a^{2}b^{2}$, $Y=-\text{（}\alpha\theta_{2}/\pi\text{）}^{2}(a^{2}+b^{2})[a^{2}(\varepsilon_{2}+1)-b^{2}(\varepsilon_{2}-1)]$, $Z=(a^{2}-b^{2})[a^{2}(\varepsilon_{2}+1)^{2}-b^{2}(\varepsilon_{2}-1)^{2}]$.

**B. Cloaking electric field and inducing magnetic field**

See Fig. B1, the bi-layer cloak consists of CAM and conductor. When a uniform electric field $\overrightarrow{E_{0}}$ is applied, the electric potential in the cylindrical coordinate system $(r,\theta,z)$ in the four regions can be written as^S1^

$\phi_{e1}=(-E_{0}r+\frac{A}{r})\cos\theta$ (B1)

$\phi_{e2}=(Br+\frac{C}{r})\cos\theta$ (B2)

$\phi_{e3}=(Dr+\frac{E}{r})\cos\theta$ (B3)

$\phi_{e4}=Fr\cos\theta$ (B4)

I

II

III

C

IV

CAM

Fig. B1. The CAM and conductor (C) bi-layer cylindrical electric cloak.

where $\phi_{e1}$, $\phi_{e2}$, $\phi_{e3}$ and $\phi_{e4}$ are the electric potential in the four regions, and $A$, $B$, $C$, $D$, $E$ and $F$ are unknown coefficients. The relation between the electric field and the electric potential is $\overrightarrow{E}=-\nabla\phi_{e}$, where $\overrightarrow{E}$ is the electric field, $\nabla=\frac{\partial}{\partial r}\overrightarrow{e_{r}}+\frac{1}{r}\frac{\partial}{\partial\theta}\overrightarrow{e_{\theta}}+\frac{\partial}{\partial z}\overrightarrow{e_{z}}$, so the electric field in the four regions can be written as

$\overrightarrow{E}_{1}=-\nabla\phi_{e1}=(E_{0}+\frac{A}{r^{2}})\cos\theta\overrightarrow{e_{r}}+(-E_{0}+\frac{A}{r^{2}})\sin\theta\overrightarrow{e_{\theta}}$ (B5)

$\overrightarrow{E}_{2}=-\nabla\phi_{e2}=(-B+\frac{C}{r^{2}})\cos\theta\overrightarrow{e_{r}}+(B+\frac{C}{r^{2}})\sin\theta\overrightarrow{e_{\theta}}$ (B6)

$\overrightarrow{E}_{3}=-\nabla\phi_{e3}=(-D+\frac{E}{r^{2}})\cos\theta\overrightarrow{e_{r}}+(D+\frac{E}{r^{2}})\sin\theta\overrightarrow{e_{\theta}}$ (B7)

$\overrightarrow{E}_{4}=-\nabla\phi_{e4}=-F\cos\theta\overrightarrow{e_{r}}+F\sin\theta\overrightarrow{e_{\theta}}$ (B8)

where $\overrightarrow{e_{r}}$ is normal unit vector, $\overrightarrow{e_{\theta}}$ is tangential unit vector. Due to the magneto-electric response of the CAM, the magnetic scalar potential is induced in the four regions

$\phi_{m1}=(A_{0}r+\frac{A_{1}}{r})\cos\theta$ (B9)

$\phi_{m2}=(B_{1}r+\frac{C_{1}}{r})\cos\theta$ (B10)

$\phi_{m3}=(D_{1}r+\frac{E_{1}}{r})\cos\theta$ (B11)

$\phi_{m4}=F_{1}r\cos\theta$ (B12)

where $\phi_{m1}$, $\phi_{m2}$, $\phi_{m3}$ and $\phi_{m4}$ are the magnetic scalar potential in the four regions, and $A_{0}$, $\text{A}_{1}$, $B_{1}$, $C_{1}$, $D_{1}$, $E_{1}$ and $F_{1}$ are unknown coefficients. Because of no physics magnetic field in region I, so $A_{0}=0$. The relation between the magnetic field and the magnetic scalar potential is $\overrightarrow{H}=-\nabla\phi_{m}$, where $\overrightarrow{H}$ is the magnetic field, the magnetic field in the four regions can be written as

$\overrightarrow{H}_{1}=-\nabla\phi_{m1}=\frac{A_{1}}{r^{2}}\cos\theta\overrightarrow{e_{r}}+\frac{A_{1}}{r^{2}}\sin\theta\overrightarrow{e_{\theta}}$ (B13)

$\overrightarrow{H}_{2}=-\nabla\phi_{m2}=(-B_{1}+\frac{C_{1}}{r^{2}})\cos\theta\overrightarrow{e_{r}}+(B_{1}+\frac{C_{1}}{r^{2}})\sin\theta\overrightarrow{e_{\theta}}$ (B14)

$\overrightarrow{H}_{3}=-\nabla\phi_{m3}=(-D_{1}+\frac{E_{1}}{r^{2}})\cos\theta\overrightarrow{e_{r}}+(D_{1}+\frac{E_{1}}{r^{2}})\sin\theta\overrightarrow{e_{\theta}}$ (B15)

$\overrightarrow{H}_{4}=-\nabla\phi_{m4}=-F_{1}\cos\theta\overrightarrow{e_{r}}+F_{1}\sin\theta\overrightarrow{e_{\theta}}$ (B16)

The electric field is continuous in the tangential direction, so

$-E_{0}+\frac{A}{a^{2}}=B+\frac{C}{a^{2}}$ (B17)

$D+\frac{E}{b^{2}}=B+\frac{C}{b^{2}}$ (B18)

$D+\frac{E}{c^{2}}=F$ (B19)

The magnetic field is continuous in the tangential direction, so

$\frac{A_{1}}{a^{2}}=B_{1}+\frac{C_{1}}{a^{2}}$ (B20)

$D_{1}+\frac{E_{1}}{b^{2}}=B_{1}+\frac{C_{1}}{b^{2}}$ (A21)

$D_{1}+\frac{E_{1}}{c^{2}}=F_{1}$ (A22)

The electric displacement is continuous in the normal direction, according to Eq. (A1), we obtain

$\varepsilon_{0}\varepsilon_{1}(E_{0}+\frac{A}{a^{2}})=\varepsilon_{0}(\varepsilon_{2}+\mu_{2}\frac{\alpha^{2}\theta_{2}^{2}}{\pi^{2}})(-B+\frac{C}{a^{2}})-\mu_{2}\sqrt{\varepsilon_{0}\mu_{0}}\frac{\alpha\theta_{2}}{\pi}(-B_{1}+\frac{C_{1}}{a^{2}})$ (A23)

$\varepsilon_{0}(\varepsilon_{2}+\mu_{2}\frac{\alpha^{2}\theta_{2}^{2}}{\pi^{2}})(-B+\frac{C}{b^{2}})-\mu_{2}\sqrt{\varepsilon_{0}\mu_{0}}\frac{\alpha\theta_{2}}{\pi}(-B_{1}+\frac{C_{1}}{b^{2}})$ $=\varepsilon_{0}(\varepsilon_{3}+\mu_{3}\frac{\alpha^{2}\theta_{3}^{2}}{\pi^{2}})(-D+\frac{E}{b^{2}})-\mu_{3}\sqrt{\varepsilon_{0}\mu_{0}}\frac{\alpha\theta_{3}}{\pi}(-D_{1}+\frac{E_{1}}{b^{2}})$ (A24)

$\varepsilon_{0}(\varepsilon_{3}+\mu_{3}\frac{\alpha^{2}\theta_{3}^{2}}{\pi^{2}})(-D+\frac{E}{c^{2}})-\mu_{3}\sqrt{\varepsilon_{0}\mu_{0}}\frac{\alpha\theta_{3}}{\pi}(-D_{1}+\frac{E_{1}}{c^{2}})=-\varepsilon_{0}\varepsilon_{4}F$ (A25)

The magnetic flux density is continuous in the normal direction, according to Eq. (A2), we obtain

$\mu_{0}\mu_{1}\frac{A_{1}}{a^{2}}=\mu_{0}\mu_{2}(-B_{1}+\frac{C_{1}}{a^{2}})-\mu_{2}\sqrt{\varepsilon_{0}\mu_{0}}\frac{\alpha\theta_{2}}{\pi}(-B+\frac{C}{a^{2}})$ (A26)

$\mu_{0}\mu_{2}(-B_{1}+\frac{C_{1}}{b^{2}})-\mu_{2}\sqrt{\varepsilon_{0}\mu_{0}}\frac{\alpha\theta_{2}}{\pi}(-B+\frac{C}{b^{2}})$ $=\mu_{0}\mu_{3}(-D_{1}+\frac{E_{1}}{b^{2}})-\mu_{3}\sqrt{\varepsilon_{0}\mu_{0}}\frac{\alpha\theta_{3}}{\pi}(-D+\frac{E}{b^{2}})$ (A27)

$\mu_{0}\mu_{3}(-D_{1}+\frac{E_{1}}{c^{2}})-\mu_{3}\sqrt{\varepsilon_{0}\mu_{0}}\frac{\alpha\theta_{3}}{\pi}(-D+\frac{E}{c^{2}})=-\mu_{0}\mu_{4}F_{1}$ (A28)

When $A=0$, the electric field is still $E_{0}$ in region I; when $F=0$, the electric field vanishes in region IV, namely, the device is an electric cloak. When $\text{A}_{1}$, $B_{1}$, $C_{1}$, $D_{1}$, $E_{1}$ and $F_{1}$ are non-zero, the magnetic field is induced by the applied electric field.

**C. Cloaking electric field of point electric charge and inducing magnetic monopole**

I

II

III

C

IV

CAM

Fig. C1. The CAM and conductor (C) bi-layer spherical electric cloak.

Fig. C2. The point electric charge $Q$ is in front of the CAM sphere.

See Fig. C1, the bi-layer spherical cloak consists of CAM and conductor. See Fig. C2, when a point electric charge $Q$ is locate at point $M$, according to the superposition principle of electric potential, the electric potential is generated by a point electric charge and CAM sphere in the spherical coordinate system $(r,\theta,\phi)$ can be written as $\phi_{e}=-Q\text{/}4\pi\varepsilon_{0}R+\sum(M_{n}r^{n}+N_{n}r^{-n-1})P_{n}(\cos\theta)$, the first term is the electric potential generated by a point electric charge, the second term is the electric potential induced by CAM sphere, the “-” in the first term implies that the zero electric potential in the original point of coordinate. See Fig. C2, utilizing Legendre mother function, when $L>r$, we have $\frac{Q}{4\pi\varepsilon_{0}R}=\frac{Q}{4\pi\varepsilon_{0}}\frac{1}{\sqrt{L^{2}+r^{2}-2Lr\cos\theta}}=\frac{Q}{4\pi\varepsilon_{0}L}\sum(\frac{r}{L})^{n}P_{n}(\cos\theta)$; when $L<r$, we have $\frac{Q}{4\pi\varepsilon_{0}R}=\frac{Q}{4\pi\varepsilon_{0}}\frac{1}{\sqrt{L^{2}+r^{2}-2Lr\cos\theta}}$ $=\frac{Q}{4\pi\varepsilon_{0}r}\sum(\frac{L}{r})^{n}P_{n}(\cos\theta)$. We take $L>r$, so the electric potential in the four regions is

$\phi_{e1}=\sum(-\frac{Q}{4\pi\varepsilon_{0}}\frac{r^{n}}{L^{n+1}}+\frac{A_{n}}{r^{n+1}})P_{n}(\cos\theta)$ (C1)

$\phi_{e2}=\sum(B_{n}r^{n}+\frac{C_{n}}{r^{n+1}})P_{n}(\cos\theta)$ (C2)

$\phi_{e3}=\sum(D_{n}r^{n}+\frac{E_{n}}{r^{n+1}})P_{n}(\cos\theta)$ (C3)

$\phi_{e4}=\sum(F_{n}r^{n})P_{n}(\cos\theta)$ (C4)

where $\phi_{e1}$, $\phi_{e2}$, $\phi_{e3}$ and $\phi_{e4}$ are the electric potential in the four regions, and $A_{n}$, $B_{n}$, $C_{n}$, $D_{n}$, $E_{n}$ and $F_{n}$ are unknown coefficients, noting that $\phi_{e2}$, $\phi_{e3}$ and $\phi_{e4}$ are the electric potential superposition of the point electric charge and the spherical cloak. The electric field in the four regions can be written as

$\overrightarrow{E}_{1}=\sum(\frac{Q}{4\pi\varepsilon_{0}}\frac{nr^{n\text{-1}}}{L^{n+1}}+\frac{(n+1)A_{n}}{r^{n+2}})P_{n}(\cos\theta)\overrightarrow{e_{r}}$ $\text{-}\sum(\text{-}\frac{Q}{4\pi\varepsilon_{0}}\frac{r^{n-1}}{L^{n+1}}+\frac{A_{n}}{r^{n+2}})\frac{\partial P_{n}(\cos\theta)}{\partial\theta}\overrightarrow{e_{\theta}}$(C5)

$\overrightarrow{E}_{2}=\sum(\text{-}nB_{n}r^{n-1}+\frac{(n+1)C_{n}}{r^{n+2}})P_{n}(\cos\theta)\overrightarrow{e_{r}}$ $\text{-}\sum(B_{n}r^{n-1}+\frac{C_{n}}{r^{n+2}})\frac{\partial P_{n}(\cos\theta)}{\partial\theta}\overrightarrow{e_{\theta}}$ (C6)

$\overrightarrow{E}_{3}=\sum(\text{-}nD_{n}r^{n-1}+\frac{(n+1)E_{n}}{r^{n+2}})P_{n}(\cos\theta)\overrightarrow{e_{r}}$ $\text{-}\sum(D_{n}r^{n-1}+\frac{E_{n}}{r^{n+2}})\frac{\partial P_{n}(\cos\theta)}{\partial\theta}\overrightarrow{e_{\theta}}$ (C7)

$\overrightarrow{E}_{4}=\text{-}\sum(nF_{n}r^{n-1})P_{n}(\cos\theta)\overrightarrow{e_{r}}$ $\text{-}\sum(F_{n}r^{n-1})\frac{\partial P_{n}(\cos\theta)}{\partial\theta}\overrightarrow{e_{\theta}}$ (C8)

Due to the magneto-electric response of the CAM, the magnetic scalar potential in the four regions is induced

$\phi_{m1}=\sum(A^{01}r^{n}+\frac{A_{n}^{1}}{r^{n+1}})P_{n}(\cos\theta)$ (C9)

$\phi_{m2}=\sum(B_{n}^{1}r^{n}+\frac{C_{n}^{1}}{r^{n+1}})P_{n}(\cos\theta)$ (C10)

$\phi_{m3}=\sum(D_{n}^{1}r^{n}+\frac{E_{n}^{1}}{r^{n+1}})P_{n}(\cos\theta)$ (C11)

$\phi_{m4}=\sum F_{n}r^{n}P_{n}(\cos\theta)$ (C12)

The magnetic field in the four regions can be written as

$\overrightarrow{H}_{1}=\text{-}\sum(\frac{(n+1)A_{n}^{1}}{r^{n+2}})P_{n}(\cos\theta)\overrightarrow{e_{r}}$ $\text{-}\sum(\frac{A_{n}^{1}}{r^{n+2}})\frac{\partial P_{n}(\cos\theta)}{\partial\theta}\overrightarrow{e_{\theta}}$ (C13)

$\overrightarrow{H}_{2}=\sum(\text{-}nB_{n}^{1}r^{n-1}+\frac{(n+1)C_{n}^{1}}{r^{n+2}})P_{n}(\cos\theta)\overrightarrow{e_{r}}$ $\text{-}\sum(B_{n}^{1}r^{n-1}+\frac{C_{n}^{1}}{r^{n+2}})\frac{\partial P_{n}(\cos\theta)}{\partial\theta}\overrightarrow{e_{\theta}}$ (C14)

$\overrightarrow{H}_{3}=\sum(\text{-}nD_{n}^{1}r^{n-1}+\frac{(n+1)E_{n}^{1}}{r^{n+2}})P_{n}(\cos\theta)\overrightarrow{e_{r}}$ $\text{-}\sum(D_{n}^{1}r^{n-1}+\frac{E_{n}^{1}}{r^{n+2}})\frac{\partial P_{n}(\cos\theta)}{\partial\theta}\overrightarrow{e_{\theta}}$ (C15)

$\overrightarrow{H}_{4}=\sum(\text{-}nF_{n}^{1}r^{n-1})P_{n}(\cos\theta)\overrightarrow{e_{r}}$ $\text{-}\sum(F_{n}^{1}r^{n-1})\frac{\partial P_{n}(\cos\theta)}{\partial\theta}\overrightarrow{e_{\theta}}$ (C16)

The electric field is continuous in the tangential direction, so

$-\frac{Q}{4\pi\varepsilon_{0}}\frac{1}{L^{n+1}}+\frac{A_{n}}{a^{2n+1}}=B_{n}+\frac{C_{n}}{a^{2n+1}}$ (C17)

$D_{n}+\frac{E_{n}}{b^{2n+1}}=B_{n}+\frac{C_{n}}{b^{2n+1}}$ (C18)

$D_{n}+\frac{E_{n}}{c^{2n+1}}=F_{n}$ (C19)

The magnetic field is continuous in the tangential direction, so

$\frac{A_{n}^{1}}{a^{2n+1}}=B_{n}^{1}+\frac{C_{n}^{1}}{a^{2n+1}}$ (C20)

$D_{n}^{1}+\frac{E_{n}^{1}}{b^{2n+1}}=B_{n}^{1}+\frac{C_{n}^{1}}{b^{2n+1}}$ (C21)

$D_{n}^{1}+\frac{E_{n}^{1}}{c^{2n+1}}=F_{n}^{1}$ (C22)

The electric displacement is continuous in the normal direction, according to Eq. (A1), we obtain

$$\varepsilon_{0}\varepsilon_{1}(\frac{Q}{4\pi\varepsilon_{0}}\frac{n}{L^{n+1}}+\frac{(n+1)A_{n}}{a^{2n+1}})$$

$=\varepsilon_{0}(\varepsilon_{2}+\mu_{2}\frac{\alpha^{2}\theta_{2}^{2}}{\pi^{2}})(\text{-}nB_{n}+\frac{(n+1)C_{n}}{a^{2n+1}})$ $-\mu_{2}\sqrt{\varepsilon_{0}\mu_{0}}\frac{\alpha\theta_{2}}{\pi}(\text{-}nB_{n}^{1}+\frac{(n+1)C_{n}^{1}}{a^{2n+1}})$ (C23)

$$\varepsilon_{0}(\varepsilon_{2}+\mu_{2}\frac{\alpha^{2}\theta_{2}^{2}}{\pi^{2}})(\text{-}nB_{n}+\frac{(n+1)C_{n}}{b^{2n+1}})$$

$$-\mu_{2}\sqrt{\varepsilon_{0}\mu_{0}}\frac{\alpha\theta_{2}}{\pi}(\text{-}nB_{n}^{1}+\frac{(n+1)C_{n}^{1}}{b^{2n+1}})$$

$=\varepsilon_{0}(\varepsilon_{3}+\mu_{3}\frac{\alpha^{2}\theta_{3}^{2}}{\pi^{2}})(\text{-}nD_{n}+\frac{(n+1)E_{n}}{b^{2n+1}})$ $-\mu_{3}\sqrt{\varepsilon_{0}\mu_{0}}\frac{\alpha\theta_{3}}{\pi}(\text{-}nD_{n}^{1}+\frac{(n+1)E_{n}^{1}}{b^{2n+1}})$ (C24)

$\varepsilon_{0}(\varepsilon_{3}+\mu_{3}\frac{\alpha^{2}\theta_{3}^{2}}{\pi^{2}})(\text{-}nD_{n}+\frac{(n+1)E_{n}}{c^{2n+1}})$ $-\mu_{3}\sqrt{\varepsilon_{0}\mu_{0}}\frac{\alpha\theta_{3}}{\pi}(\text{-}nD_{n}^{1}+\frac{(n+1)E_{n}^{1}}{c^{2n+1}})$ $=\text{-}\varepsilon_{0}\varepsilon_{4}nF_{n}$(C25)

The magnetic flux density is continuous in the normal direction, according to Eq. (A2), we obtain

$\mu_{0}\mu_{1}(\frac{(n+1)A_{n}^{1}}{a^{2n+1}})=\mu_{0}\mu_{2}(\text{-}nB_{n}^{1}+\frac{(n+1)C_{n}^{1}}{a^{2n+1}})$ $-\mu_{2}\sqrt{\varepsilon_{0}\mu_{0}}\frac{\alpha\theta_{2}}{\pi}(\text{-}nB_{n}+\frac{(n+1)C_{n}}{a^{2n+1}})$ (C26)

$\mu_{0}\mu_{2}(\text{-}nB_{n}^{1}+\frac{(n+1)C_{n}^{1}}{b^{2n+1}})$ $-\mu_{2}\sqrt{\varepsilon_{0}\mu_{0}}\frac{\alpha\theta_{2}}{\pi}(\text{-}nB_{n}+\frac{(n+1)C_{n}}{b^{2n+1}})$ $=\mu_{0}\mu_{3}(\text{-}nD_{n}^{1}+\frac{(n+1)E_{n}^{1}}{b^{2n+1}})$ $-\mu_{3}\sqrt{\varepsilon_{0}\mu_{0}}\frac{\alpha\theta_{3}}{\pi}(\text{-}nD_{n}+\frac{(n+1)E_{n}}{b^{2n+1}})$ (C27)

$\mu_{0}\mu_{3}(\text{-}nD_{n}^{1}+\frac{(n+1)E_{n}^{1}}{c^{2n+1}})$ $-\mu_{3}\sqrt{\varepsilon_{0}\mu_{0}}\frac{\alpha\theta_{3}}{\pi}(\text{-}nD_{n}+\frac{(n+1)E_{n}}{c^{2n+1}})$ $=\text{-}\mu_{0}\mu_{4}nF_{n}^{1}$ (C28)

When $A_{n}=0$, the electric field in region I is generated only by the point electric charge; when $F_{n}=0$, the electric field vanishes in region IV, namely, the device is an electric cloak. When $\text{A}_{\text{n}}^{1}$, $\text{B}_{\text{n}}^{1}$, $\text{C}_{\text{n}}^{1}$, $\text{D}_{\text{n}}^{1}$, $\text{E}_{\text{n}}^{1}$ and $\text{F}_{\text{n}}^{1}$ are non-zero, the magnetic field is induced by the point electric charge.

According to Eq. (C9)-(C12) and magnetic charge formulation^S2^, the interesting thing emerges, namely, many monopoles are induced in the space. For example, neglecting infinitesimal of higher order, according to Eq. (C9), we obtain coefficient $A_{n}^{1}=-\frac{\alpha\varepsilon_{1}}{\varepsilon_{2}}\sqrt{\frac{\varepsilon_{0}}{\mu_{0}}}\frac{Q}{4\pi\varepsilon_{0}}\frac{n}{2n+1}\frac{a^{2n+1}}{L^{n+1}}$, so, the magnetic scalar potential in region I

$$\phi_{m1}=-\frac{\alpha\varepsilon_{1}}{\varepsilon_{2}}\sqrt{\frac{\varepsilon_{0}}{\mu_{0}}}\frac{Q}{4\pi\varepsilon_{0}}\sum(\frac{n}{2n+1}\frac{a^{2n+1}}{L^{n+1}r^{n+1}})P_{n}(\cos\theta)$$

$=-\frac{\alpha\varepsilon_{1}}{\varepsilon_{2}}\sqrt{\frac{\varepsilon_{0}}{\mu_{0}}}\frac{Q}{4\pi\varepsilon_{0}}\frac{a}{L}\sum(\frac{n}{2n+1}\frac{s^{n}}{r^{n+1}})P_{n}(\cos\theta)$ ($a^{2}=sL$)

$$=-\frac{\alpha\varepsilon_{1}}{\varepsilon_{2}}\sqrt{\frac{\varepsilon_{0}}{\mu_{0}}}\frac{Q}{4\pi\varepsilon_{0}}\frac{a}{L}\sum(\frac{s^{n}}{r^{n+1}})P_{n}(\cos\theta)$$

$$+\frac{\alpha\varepsilon_{1}}{\varepsilon_{2}}\sqrt{\frac{\varepsilon_{0}}{\mu_{0}}}\frac{Q}{4\pi\varepsilon_{0}}\frac{a}{L}\sum(\frac{n+1}{2n+1}\frac{s^{n}}{r^{n+1}})P_{n}(\cos\theta)$$

$$=-\frac{\alpha\varepsilon_{1}}{\varepsilon_{2}}\sqrt{\frac{\varepsilon_{0}}{\mu_{0}}}\frac{Q}{4\pi\varepsilon_{0}}\frac{a}{L}\frac{1}{\sqrt{r^{2}+s^{2}-2rs\cos\theta}}$$

$$+\frac{\alpha\varepsilon_{1}}{\varepsilon_{2}}\sqrt{\frac{\varepsilon_{0}}{\mu_{0}}}\frac{Q}{4\pi\varepsilon_{0}}\frac{a}{L}\sum(\frac{n+1}{2n+1}\frac{s^{n}}{r^{n+1}})P_{n}(\cos\theta)$$

The first term of the right tell us that there is a monopole in inversion point (point $\text{D}$ in Fig. C2, $\text{OD}=\text{s}$), the magnetic scalar potential is induced by point electric charge, the monopole charge may be positive or negative, the positive or negative is decided by angle $\theta$.

**Supplementary Text**

**D. Cloaking magnetic field and inducing electric field with bi-layer sphere**

I

II

III

SC

IV

CAM

Fig. D1. The CAM and superconductor (SC) bi-layer spherical magnetic cloak.

When a uniform magnetic field $\overrightarrow{H_{0}}$ is applied to the spherical cloak, the magnetic scalar potential in the spherical coordinate system $(r,\theta,\phi)$ in the four regions can be written as

$\phi_{m1}=(-H_{0}r+\frac{A}{r^{2}})\cos\theta$ (D1)

$\phi_{m2}=(Br+\frac{C}{r^{2}})\cos\theta$ (D2)

$\phi_{m3}=(Dr+\frac{E}{r^{2}})\cos\theta$ (D3)

$\phi_{m4}=Fr\cos\theta$ (D4)

where $\phi_{m1}$, $\phi_{m2}$, $\phi_{m3}$ and $\phi_{m4}$ are the magnetic scalar potential in the four regions, and $A$, $B$, $C$, $D$, $E$ and $F$ are unknown coefficients. The relation between the magnetic field and the magnetic scalar potential is $\overrightarrow{H}=-\nabla\phi_{m}$, where $\nabla=\frac{\partial}{\partial r}\overrightarrow{e_{r}}+\frac{1}{r}\frac{\partial}{\partial\theta}\overrightarrow{e_{\theta}}+\frac{1}{r\sin\theta}\frac{\partial}{\partial\varphi}\overrightarrow{e_{\varphi}}$, $\overrightarrow{H}$ is the magnetic field, so the magnetic field in the four regions can be written as

$\overrightarrow{H}_{1}=-\nabla\phi_{m1}=(H_{0}+\frac{2A}{r^{3}})\cos\theta\overrightarrow{e_{r}}+(-H_{0}+\frac{A}{r^{3}})\sin\theta\overrightarrow{e_{\theta}}$ (D5)

$\overrightarrow{H}_{2}=-\nabla\phi_{m2}=(-B+\frac{2C}{r^{3}})\cos\theta\overrightarrow{e_{r}}+(B+\frac{C}{r^{3}})\sin\theta\overrightarrow{e_{\theta}}$ (D6)

$\overrightarrow{H}_{3}=-\nabla\phi_{m3}=(-D+\frac{2E}{r^{3}})\cos\theta\overrightarrow{e_{r}}+(D+\frac{E}{r^{3}})\sin\theta\overrightarrow{e_{\theta}}$ (D7)

$\overrightarrow{H}_{4}=-\nabla\phi_{m4}=-F\cos\theta\overrightarrow{e_{r}}+F\sin\theta\overrightarrow{e_{\theta}}$ (D8)

Due to the magneto-electric response of the CAM, the electric potential in the four regions is induced

$\phi_{e1}=(A_{0}r+\frac{A_{1}}{r^{2}})\cos\theta$ (D9)

$\phi_{e2}=(B_{1}r+\frac{C_{1}}{r^{2}})\cos\theta$ (D10)

$\phi_{e3}=(D_{1}r+\frac{E_{1}}{r^{2}})\cos\theta$ (D11)

$\phi_{e4}=F_{1}r\cos\theta$ (D12)

where $\phi_{e1}$, $\phi_{e2}$, $\phi_{e3}$ and $\phi_{e4}$ are the electric potential in the four regions, and $A_{0}$, $A_{1}$, $B_{1}$, $C_{1}$, $D_{1}$, $E_{1}$ and $F_{1}$ are unknown coefficients. Because of no physics electric field in region I, so $A_{0}=0$. The relation between the electric field and the electric potential is $\overrightarrow{E}=-\nabla\phi_{e}$, where $\overrightarrow{E}$ is the electric field, the electric field in the four regions can be written as

$\overrightarrow{E}_{1}=-\nabla\phi_{e1}=\frac{2A_{1}}{r^{3}}\cos\theta\overrightarrow{e_{r}}+\frac{A_{1}}{r^{3}}\sin\theta\overrightarrow{e_{\theta}}$ (D13)

$\overrightarrow{E}_{2}=-\nabla\phi_{e2}=(-B_{1}+\frac{2C_{1}}{r^{3}})\cos\theta\overrightarrow{e_{r}}+(B_{1}+\frac{C_{1}}{r^{3}})\sin\theta\overrightarrow{e_{\theta}}$ (D14)

$\overrightarrow{E}_{3}=-\nabla\phi_{e3}=(-D_{1}+\frac{2E_{1}}{r^{3}})\cos\theta\overrightarrow{e_{r}}+(D_{1}+\frac{E_{1}}{r^{3}})\sin\theta\overrightarrow{e_{\theta}}$ (D15)

$\overrightarrow{E}_{4}=-\nabla\phi_{e4}=-F_{1}\cos\theta\overrightarrow{e_{r}}+F_{1}\sin\theta\overrightarrow{e_{\theta}}$ (D16)

The magnetic field is continuous in the tangential direction

$-H_{0}+\frac{A}{a^{3}}=B+\frac{C}{a^{3}}$ (D17)

$D+\frac{E}{b^{3}}=B+\frac{C}{b^{3}}$ (D18)

$D+\frac{E}{c^{3}}=F$ (D19)

The electric field is continuous in the tangential direction

$\frac{A_{1}}{a^{3}}=B_{1}+\frac{C_{1}}{a^{3}}$ (D20)

$D_{1}+\frac{E_{1}}{b^{3}}=B_{1}+\frac{C_{1}}{b^{3}}$ (D21)

$D_{1}+\frac{E_{1}}{c^{3}}=F_{1}$ (D22)

The electric displacement is continuous in the normal direction, according to Eq. (A1), we obtain

$\varepsilon_{0}\varepsilon_{1}\frac{2A_{1}}{a^{3}}=\varepsilon_{0}(\varepsilon_{2}+\mu_{2}\frac{\alpha^{2}\theta_{2}^{2}}{\pi^{2}})(-B_{1}+\frac{2C_{1}}{a^{3}})-\mu_{2}\sqrt{\varepsilon_{0}\mu_{0}}\frac{\alpha\theta_{2}}{\pi}(-B+\frac{2C}{a^{3}})$ (D23)

$\varepsilon_{0}(\varepsilon_{2}+\mu_{2}\frac{\alpha^{2}\theta_{2}^{2}}{\pi^{2}})(-B_{1}+\frac{2C_{1}}{b^{3}})-\mu_{2}\sqrt{\varepsilon_{0}\mu_{0}}\frac{\alpha\theta_{2}}{\pi}(-B+\frac{2C}{b^{3}})$ $=\varepsilon_{0}(\varepsilon_{3}+\mu_{3}\frac{\alpha^{2}\theta_{3}^{2}}{\pi^{2}})(-D_{1}+\frac{2E_{1}}{b^{3}})-\mu_{3}\sqrt{\varepsilon_{0}\mu_{0}}\frac{\alpha\theta_{3}}{\pi}(-D+\frac{2E}{b^{3}})$ (D24)

$\varepsilon_{0}(\varepsilon_{3}+\mu_{3}\frac{\alpha^{2}\theta_{3}^{2}}{\pi^{2}})(-D_{1}+\frac{2E_{1}}{c^{3}})-\mu_{3}\sqrt{\varepsilon_{0}\mu_{0}}\frac{\alpha\theta_{3}}{\pi}(-D+\frac{2E}{c^{3}})=-\varepsilon_{0}\varepsilon_{4}F_{1}$ (D25)

The magnetic flux density is continuous in the normal direction, according to Eq. (A2), we obtain

$\mu_{0}\mu_{1}(H_{0}+\frac{2A}{a^{3}})=\mu_{0}\mu_{2}(-B+\frac{2C}{a^{3}})-\mu_{2}\sqrt{\varepsilon_{0}\mu_{0}}\frac{\alpha\theta_{2}}{\pi}(-B_{1}+\frac{2C_{1}}{a^{3}})$ (D26)

$\mu_{0}\mu_{2}(-B+\frac{2C}{b^{3}})-\mu_{2}\sqrt{\varepsilon_{0}\mu_{0}}\frac{\alpha\theta_{2}}{\pi}(-B_{1}+\frac{2C_{1}}{b^{3}})$ $=\mu_{0}\mu_{3}(-D+\frac{2E}{b^{3}})-\mu_{3}\sqrt{\varepsilon_{0}\mu_{0}}\frac{\alpha\theta_{3}}{\pi}(-D_{1}+\frac{2E_{1}}{b^{3}})$ (D27)

$\mu_{0}\mu_{3}(-D+\frac{2E}{c^{3}})-\mu_{3}\sqrt{\varepsilon_{0}\mu_{0}}\frac{\alpha\theta_{3}}{\pi}(-D_{1}+\frac{2E_{1}}{c^{3}})=-\mu_{0}\mu_{4}F$ (D28)


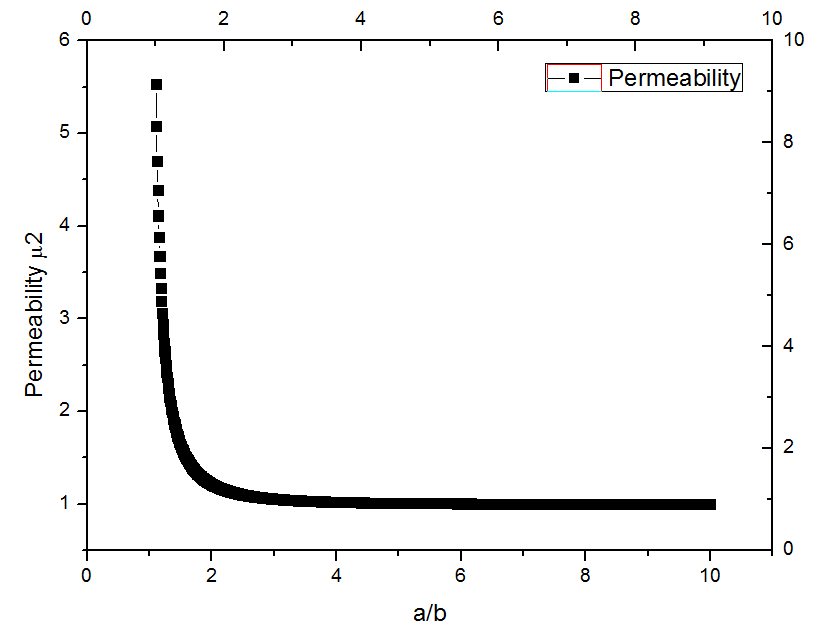


Fig. D2. The relation between the permeability $\mu_{2}$ and the radii ratios $a/b$.

When $A=0$, $F=0$, the magnetic field is cloaked. When $A_{1}$, $B_{1}$, $C_{1}$, $D_{1}$, $E_{1}$ and $F_{1}$ are non-zero, the electric field is induced. Supposed that the inner layer is a superconductor, namely, $\theta_{3}=0$, $\mu_{3}=0$, we take the permittivity of the superconductor $\varepsilon_{3}=1$, solving Eq. (D17) - Eq. (D28), we can obtain the condition of the cloak, the relations between the size parameters and material parameters are

$\mu_{2}=\frac{(2a^{3}+b^{3})[a^{3}(2\varepsilon_{2}+1)(\varepsilon_{2}+2)-2b^{3}(\varepsilon_{2}-1)^{2}]}{X+Y+Z}$ (29)

where$X=\text{9（}\alpha\theta_{2}/\pi\text{）}^{2}a^{3}b^{3}$,$Y=-\text{（}\alpha\theta_{2}/\pi\text{）}^{2}(2a^{3}+b^{3})[a^{3}(2\varepsilon_{2}+1)-2b^{3}(\varepsilon_{2}-1)]$ and $Z=2(a^{3}-b^{3})[a^{3}(2\varepsilon_{2}+1)(\varepsilon_{2}+2)-2b^{3}(\varepsilon_{2}-1)^{2}]$. Fig. D2 shows the relation between the radii ratios $a/b$ and permeability$\mu_{2}$, the magneto-electric polarizability $\theta_{2}=\pi$, $\varepsilon_{2}=2$, $\alpha=1/137$. Fig. D2 shows the conditions of the cloak, for example, if $a/b=2$, $\mu_{2}=1.214$, then $A=0$, $F=0$; if $a/b=2.5$, $\mu_{2}=1.103$, then $A=0$, $F=0$.


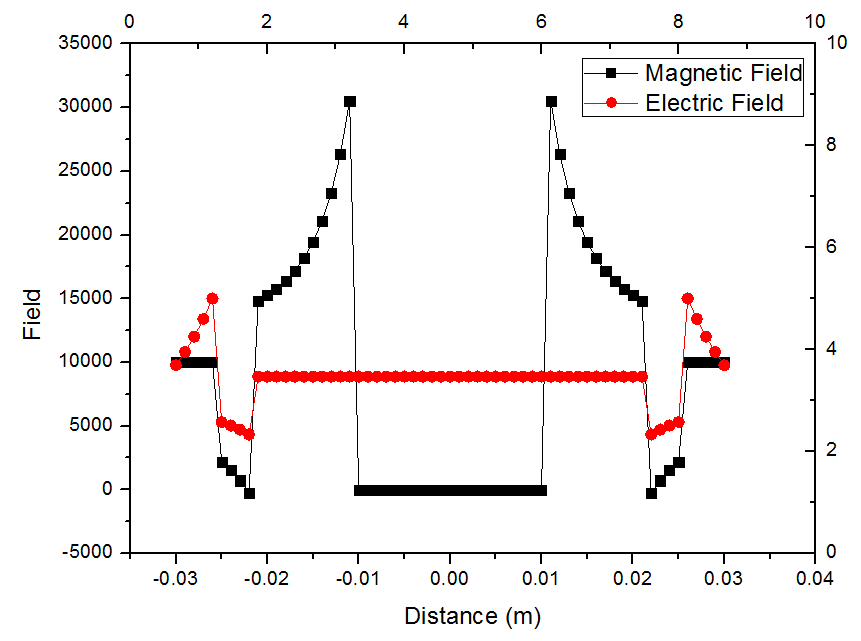


Fig. D3. The distributions of the magnetic field and electric field in the center line ($\text{y}=0$,$\text{z}=0$).

Fig. D3 shows the distributions of the magnetic field and the electric field in the center line ($\text{y}=0$, $\text{z}=0$), the black lines stand for the magnetic field in the center line, and the red lines stand for the electric field in the center line, the parameters are $a=0.025m$, $b=0.0222m$, $c=0.0\text{10m}$, $\mu_{2}=4.556$, $H_{0}=10000A/m$. When $r>0.025m$, the magnetic field is still $H_{0}=10000A/m$, yet the induced electric fields vary with space; when $0.0222m<r<0.02\text{5m}$, the magnetic field and the induced electric field both vary with space; when $0.0\text{10m}<r<0.0\text{222m}$, the magnetic fields vary with space, yet the induced electric field is uniform, $\text{E}_{3}=8880\text{V}/m$; when $r<0.0\text{10m}$, the magnetic field $\text{H}_{4}=0$, the induced electric field is uniform, and the electric field is $\text{E}_{4}=8880\text{V}/m$. Namely, the CAM and superconductor bi-layer sphere cloaked applied magnetic field and induced electric field. We noted that the electric field is also uniform in the superconductor ($0.010m<r<0.0222m$), this is because we take the permittivity of the superconductor $\varepsilon_{3}=1$, when $\varepsilon_{3}\neq1$, the electric field is not uniform in the superconductor.

**E. Cloaking electric field and inducing magnetic field with CAM doped electrets material**

The calculation results show that it is impossible to obtain a cloak when the inner layer and outer layer are both the CAM, yet when the bi-layer CAMs are doped with electret material, we can design electric cloak. See Fig. E1, the bi-layer cloak consists of CAM doped with electret material, the outer layer is doped with polytetrafluoroethylene (Teflon) electret material, and the inner layer is doped with polypropylene (PP) electret material. The permittivity and permeability of the outer layer are $\varepsilon_{2}$ and $\mu_{2}$, respectively, the permittivity and permeability of the inner layer are $\varepsilon_{3}$ and $\mu_{3}$, respectively. The outer radius and inner radius of the outer layer are $a$ and $b$, respectively, the outer radius and inner radius of the inner layer are $b$ and $c$, respectively. The intrinsic polarization intensity of the outer layer and inner layer are $\overrightarrow{P}_{2}$ and $\overrightarrow{P}_{3}$, respectively, and the direction of the intrinsic polarization intensity is parallel to one of the diameters of the CAM cylinder (black arrows in Fig. E1).

I

II

III

CAM+PP

CAM+Teflon

IV

Fig. E1. The bi-layer CAM doped with electret cylindrical electric cloak.

When a uniform electric field $\overrightarrow{E_{0}}$ is applied, the electric potential in the cylindrical coordinate system $(r,\theta,z)$ in the four regions can be written as

$\phi_{e1}=(-E_{0}r+\frac{A}{r})\cos\theta$ (E1)

$\phi_{e2}=(Br+\frac{C}{r})\cos\theta$ (E2)

$\phi_{e3}=(Dr+\frac{E}{r})\cos\theta$ (E3)

$\phi_{e4}=Fr\cos\theta$ (E4)

where $\phi_{e1}$, $\phi_{e2}$, $\phi_{e3}$ and $\phi_{e4}$ are the electric potential in the four regions, and $A$, $B$, $C$, $D$, $E$ and $F$ are unknown coefficients.

When the applied electric field $\overrightarrow{E}\neq0$, the total polarization intensity $\overrightarrow{P}_{total}=\overrightarrow{P^{'}}+\overrightarrow{P}=\chi_{e}\varepsilon_{0}\overrightarrow{E}+\overrightarrow{P}$, so

$\overrightarrow{D}=\varepsilon_{0}\overrightarrow{E}+\overrightarrow{P}_{total}$ $=\varepsilon_{0}\overrightarrow{E}+\chi_{e}\varepsilon_{0}\overrightarrow{E}+\overrightarrow{P}=\varepsilon\varepsilon_{0}\overrightarrow{E}+\overrightarrow{P}$ (E5)

where $\overrightarrow{D}$ is the electric displacement vector, $\overrightarrow{P}$ is the intrinsic polarization intensity of the electret, $\overrightarrow{P^{'}}$ is the polarization intensity induced by the applied electric field, $\chi_{e}$ is the electric susceptibility, and $\varepsilon=1+\chi_{e}$ is the relative permittivity. The relation between the electric field and the electric potential is $\overrightarrow{E}=-\nabla\phi_{e}$, so the electric field in the four regions can be written as

$\overrightarrow{E}_{1}=-\nabla\phi_{e1}=(E_{0}+\frac{A}{r^{2}})\cos\theta\overrightarrow{e_{r}}+(-E_{0}+\frac{A}{r^{2}})\sin\theta\overrightarrow{e_{\theta}}$ (E6)

$\overrightarrow{E}_{2}=-\nabla\phi_{e2}=(-B+\frac{C}{r^{2}})\cos\theta\overrightarrow{e_{r}}+(B+\frac{C}{r^{2}})\sin\theta\overrightarrow{e_{\theta}}$ (E7)

$\overrightarrow{E}_{3}=-\nabla\phi_{e3}=(-D+\frac{E}{r^{2}})\cos\theta\overrightarrow{e_{r}}+(D+\frac{E}{r^{2}})\sin\theta\overrightarrow{e_{\theta}}$ (E8)

$\overrightarrow{E}_{4}=-\nabla\phi_{e4}=-F\cos\theta\overrightarrow{e_{r}}+F\sin\theta\overrightarrow{e_{\theta}}$ (E9)

Due to the magneto-electric response of the CAM, the magnetic scalar potential in the four regions are

$\phi_{m1}=(A_{0}r+\frac{A_{1}}{r})\cos\theta$ (E10)

$\phi_{m2}=(B_{1}r+\frac{C_{1}}{r})\cos\theta$ (E11)

$\phi_{m3}=(D_{1}r+\frac{E_{1}}{r})\cos\theta$ (E12)

$\phi_{m4}=F_{1}r\cos\theta$ (E13)

where $\phi_{m1}$, $\phi_{m2}$, $\phi_{m3}$ and $\phi_{m4}$ are the magnetic scalar potential in the four regions, and $A_{1}$, $B_{1}$, $C_{1}$, $D_{1}$, $E_{1}$ and $F_{1}$ are unknown coefficients. Because of no physics magnetic field in region I, so $A_{0}=0$. The relation between the magnetic field and the magnetic scalar potential is $\overrightarrow{H}=-\nabla\phi_{m}$, so the magnetic field in the four regions can be written as

$\overrightarrow{H}_{1}=-\nabla\phi_{m1}=\frac{A_{1}}{r^{2}}\cos\theta\overrightarrow{e_{r}}+\frac{A_{1}}{r^{2}}\sin\theta\overrightarrow{e_{\theta}}$ (E14)

$\overrightarrow{H}_{2}=-\nabla\phi_{m2}=(-B_{1}+\frac{C_{1}}{r^{2}})\cos\theta\overrightarrow{e_{r}}+(B_{1}+\frac{C_{1}}{r^{2}})\sin\theta\overrightarrow{e_{\theta}}$ (E15)

$\overrightarrow{H}_{3}=-\nabla\phi_{m3}=(-D_{1}+\frac{E_{1}}{r^{2}})\cos\theta\overrightarrow{e_{r}}+(D_{1}+\frac{E_{1}}{r^{2}})\sin\theta\overrightarrow{e_{\theta}}$ (E16)

$\overrightarrow{H}_{4}=-\nabla\phi_{m4}=-F_{1}\cos\theta\overrightarrow{e_{r}}+F_{1}\sin\theta\overrightarrow{e_{\theta}}$ (E17)

The electric field is continuous in the tangential direction

$-E_{0}+\frac{A}{a^{2}}=B+\frac{C}{a^{2}}$ (E18)

$D+\frac{E}{b^{2}}=B+\frac{C}{b^{2}}$ (E19)

$D+\frac{E}{c^{2}}=F$ (E20)

The magnetic field is continuous in the tangential direction

$\frac{A_{1}}{a^{2}}=B_{1}+\frac{C_{1}}{a^{2}}$ (E21)

$D_{1}+\frac{E_{1}}{b^{2}}=B_{1}+\frac{C_{1}}{b^{2}}$ (E22)

$D_{1}+\frac{E_{1}}{c^{2}}=F_{1}$ (E23)

The magnetic flux density is continuous in the normal direction, we obtain

$\mu_{0}\mu_{1}\frac{A_{1}}{a^{2}}=\mu_{0}\mu_{2}(-B_{1}+\frac{C_{1}}{a^{2}})-\mu_{2}\sqrt{\varepsilon_{0}\mu_{0}}\frac{\alpha\theta_{2}}{\pi}(-B+\frac{C}{a^{2}})$ (E24)

$\mu_{0}\mu_{2}(-B_{1}+\frac{C_{1}}{b^{2}})-\mu_{2}\sqrt{\varepsilon_{0}\mu_{0}}\frac{\alpha\theta_{2}}{\pi}(-B+\frac{C}{b^{2}})$ $=\mu_{0}\mu_{3}(-D_{1}+\frac{E_{1}}{b^{2}})-\mu_{3}\sqrt{\varepsilon_{0}\mu_{0}}\frac{\alpha\theta_{3}}{\pi}(-D+\frac{E}{b^{2}})$ (E25)

$\mu_{0}\mu_{3}(-D_{1}+\frac{E_{1}}{c^{2}})-\mu_{3}\sqrt{\varepsilon_{0}\mu_{0}}\frac{\alpha\theta_{3}}{\pi}(-D+\frac{E}{c^{2}})=-\mu_{0}\mu_{4}F_{1}$ (E26)

The electric displacement is continuous in the normal direction, combine Eq. (A1) and Eq. (E5), we obtain

$\varepsilon_{0}\varepsilon_{1}(E_{0}+\frac{A}{a^{2}})=\varepsilon_{0}(\varepsilon_{2}+\mu_{2}\frac{\alpha^{2}\theta_{2}^{2}}{\pi^{2}})(-B+\frac{C}{a^{2}})+P_{2}-\mu_{2}\sqrt{\varepsilon_{0}\mu_{0}}\frac{\alpha\theta_{2}}{\pi}(-B_{1}+\frac{C_{1}}{a^{2}})$ (E27)

$\varepsilon_{0}(\varepsilon_{2}+\mu_{2}\frac{\alpha^{2}\theta_{2}^{2}}{\pi^{2}})(-B+\frac{C}{b^{2}})+P_{2}-\mu_{2}\sqrt{\varepsilon_{0}\mu_{0}}\frac{\alpha\theta_{2}}{\pi}(-B_{1}+\frac{C_{1}}{b^{2}})$ $=\varepsilon_{0}(\varepsilon_{3}+\mu_{3}\frac{\alpha^{2}\theta_{3}^{2}}{\pi^{2}})(-D+\frac{E}{b^{2}})+P_{3}-\mu_{3}\sqrt{\varepsilon_{0}\mu_{0}}\frac{\alpha\theta_{3}}{\pi}(-D_{1}+\frac{E_{1}}{b^{2}})$ (E28)

$\varepsilon_{0}(\varepsilon_{3}+\mu_{3}\frac{\alpha^{2}\theta_{3}^{2}}{\pi^{2}})(-D+\frac{E}{c^{2}})+P_{3}-\mu_{3}\sqrt{\varepsilon_{0}\mu_{0}}\frac{\alpha\theta_{3}}{\pi}(-D_{1}+\frac{E_{1}}{c^{2}})=-\varepsilon_{0}\varepsilon_{3}F$ (E29)

Noting that, the normal polarization intensity $\overrightarrow{P}_{r}=\overrightarrow{P}\cdot\overrightarrow{e}_{r}=P\cos\theta$. When $A=0$, the electric field is still $E_{0}$ in region I, when $F=0$, the electric field vanishes in region IV, when $A_{1}$, $B_{1}$, $C_{1}$, $D_{1}$, $E_{1}$ and $F_{1}$ are non-zero, the magnetic field is induced by the applied electric field.


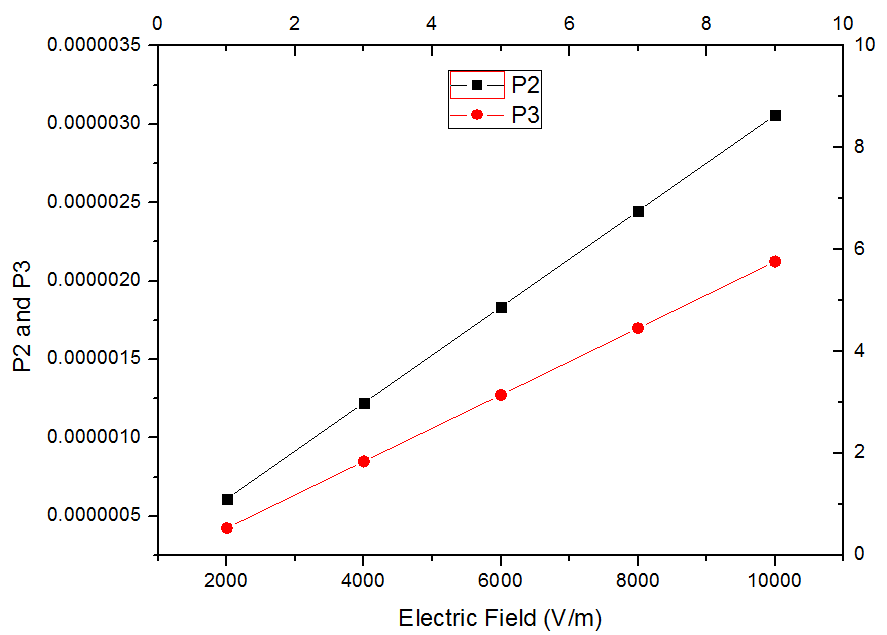


Fig. E2. The relations between the intrinsic polarization intensity and the applied electric field $E_{0}$


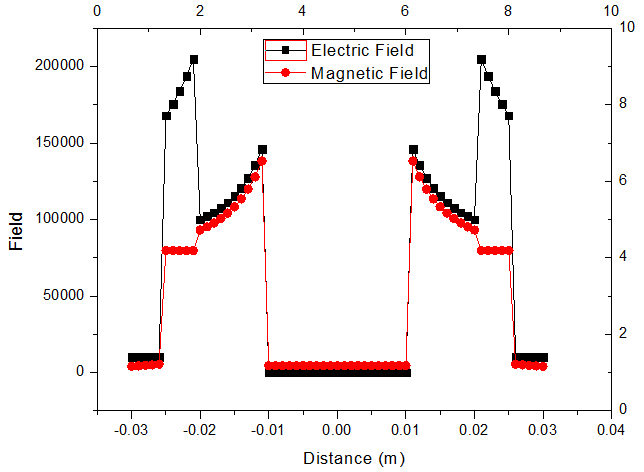


Fig. E3. The distributions of the electric field and magnetic field in the center line ($\text{y}=0$,$\text{z}=0$), the magnetic fields are 50000 times actual magnetic field.

The bi-layer CAM cylinder doped with different electret can cloak different applied electric field, Fig. E2 shows the relations between the intrinsic polarization intensity and the applied electric field $E_{0}$, the magneto-electric polarizability $\theta_{2}=\pi$, $\theta_{3}=\pi$; the other parameters are $\varepsilon_{1}=1$, $\varepsilon_{2}=2$, $\varepsilon_{3}=1.5$, $\varepsilon_{4}=1$, $\mu_{1}=1$, $\mu_{2}=2$, $\mu_{3}=1.5$, $\mu_{4}=1$; the fine-structure constant $\alpha=1/137$, the permittivity and permeability of the free space $\varepsilon_{0}=8.85\times10^{12}F/m$, $\mu_{0}=4\pi\times10^{-7}H/m$, respectively, the size parameter $a=0.0\text{25m}$, $b=0.02\text{0m}$, $c=0.01\text{0m}$. The different intrinsic polarization intensity can cloak different applied electric field, for example, when the intrinsic polarization intensity are $P_{2}=3.058\times10^{-6}C/m^{2}$ are $P_{3}=-2.124\times10^{-6}C/m^{2}$, we can cloak the applied electric field $E_{0}=10000V/m$; when the intrinsic polarization intensity are $P_{2}=1.835\times10^{-6}C/m^{2}$ and $P_{3}=-1.274\times10^{-6}C/m^{2}$, we can cloak the applied electric field $E_{0}=6000V/m$.

Fig. E3 shows the distributions of the electric field and the magnetic field in the center line ($\text{y}=0$, $\text{z}=0$), the black lines stand for the electric field in the center line, the red lines stand for the induced magnetic field in the center line, the intrinsic polarization intensity $P_{2}=3.058\times10^{-6}C/m^{2}$, $P_{3}=-2.124\times10^{-6}C/m^{2}$; the magneto-electric polarizability $\theta_{2}=\pi$, $\theta_{3}=\pi$; the other parameters are $\varepsilon_{1}=1$, $\varepsilon_{2}=2$, $\varepsilon_{3}=1.5$, $\varepsilon_{4}=1$, $\mu_{1}=1$, $\mu_{2}=2$, $\mu_{3}=1.5$, $\mu_{4}=1$; the applied electric field $E_{0}=10000V/m$, the fine-structure constant $\alpha=1/137$, the permittivity and permeability of the free space $\varepsilon_{0}=8.85\times10^{12}F/m$, $\mu_{0}=4\pi\times10^{-7}H/m$, respectively. $a=0.0\text{25m}$, $b=0.02\text{0m}$, $c=0.01\text{0m}$. When $r>0.025m$, the electric field is still $E_{0}=10000V/m$, yet the induced magnetic field varies with space; when $0.020m<r<0.025m$, the electric field and the induced magnetic field vary with space; when $0.010m<r<0.02\text{0m}$, the electric field and the induced magnetic field also vary with space; when $r<0.01\text{0m}$, the electric field is $E_{4}=0$, the induced magnetic field $H_{4}=0.0916A/m$ is uniform. Namely, the bi-layer CAM cylinder cloaked applied electric field and induced magnetic field.

**F. Cloaking magnetic field inducing electric field with CAM doped NdFeB permanent magnetic material**

See Fig. F1, the bi-layer magnetic cloak consists of CAM doped with Neodymium Iron Boron (NdFeB) permanent magnetic material, the outer layer is made of CAM doped with NdFeB (N50), and the inner layer is made of CAM doped with NdFeB (N35). The permittivity and permeability of the outer layer are $\varepsilon_{2}$ and $\mu_{2}$, respectively, the permittivity and permeability of the inner layer are $\varepsilon_{3}$ and $\mu_{3}$, respectively. The outer radius and inner radius of the outer layer are $a$ and $b$, respectively, the outer radius and inner radius of the inner layer are $b$ and $c$, respectively. The intrinsic magnetization intensity of the outer layer and inner layer is $\overrightarrow{M}_{2}$ and $\overrightarrow{M}_{3}$, respectively, and the direction of the intrinsic magnetization intensity is parallel to one of the diameters of the CAM cylinder (black arrows in Fig. F1).

I

II

III

CAM+N35

CAM+N50

IV

Fig. F1. The bi-layer CAM with NFeB cylindrical magnetic cloak.

When a uniform magnetic field $\overrightarrow{H_{0}}$ is applied, the magnetic scalar potential in the cylindrical coordinate system $(r,\theta,z)$ in the four regions can be written as

$\phi_{m1}=(-H_{0}r+\frac{A}{r})\cos\theta$ (F1)

$\phi_{m2}=(Br+\frac{C}{r})\cos\theta$ (F2)

$\phi_{m3}=(Dr+\frac{E}{r})\cos\theta$ (F3)

$\phi_{m4}=Fr\cos\theta$ (F4)

where $\phi_{m1}$, $\phi_{m2}$, $\phi_{m3}$ and $\phi_{m4}$ are the magnetic scalar potential in the four regions, and $A$, $B$, $C$, $D$, $E$ and $F$ are unknown coefficients. When the applied magnetic field $\overrightarrow{H}\neq0$, the total magnetization intensity $\overrightarrow{M}_{total}=\overrightarrow{M^{'}}+\overrightarrow{M}=\chi_{m}\overrightarrow{H}+\overrightarrow{M}$, so

$\overrightarrow{B}=\mu_{0}(\overrightarrow{H}+\overrightarrow{M})$ $=\mu_{0}(\overrightarrow{H}+\chi_{m}\overrightarrow{H}+\overrightarrow{M})=\mu\mu_{0}\overrightarrow{H}+\mu_{0}\overrightarrow{M}$ (F5)

where $\overrightarrow{B}$ is the magnetic flux density, $\overrightarrow{M}$ is the intrinsic magnetization intensity, $\overrightarrow{M^{'}}$ is the magnetization intensity induced by the applied magnetic field, $\chi_{m}$ is the magnetic susceptibility.

The relation between the magnetic field and the magnetic scalar potential is $\overrightarrow{H}=-\nabla\phi_{m}$, where $\overrightarrow{H}$ is the magnetic field, $\nabla=\frac{\partial}{\partial r}\overrightarrow{e_{r}}+\frac{1}{r}\frac{\partial}{\partial\theta}\overrightarrow{e_{\theta}}+\frac{\partial}{\partial z}\overrightarrow{e_{z}}$, so the magnetic field in the four regions can be written as

$\overrightarrow{H}_{1}=-\nabla\phi_{m1}=(H_{0}+\frac{A}{r^{2}})\cos\theta\overrightarrow{e_{r}}+(-H_{0}+\frac{A}{r^{2}})\sin\theta\overrightarrow{e_{\theta}}$ (F6)

$\overrightarrow{H}_{2}=-\nabla\phi_{m2}=(-B+\frac{C}{r^{2}})\cos\theta\overrightarrow{e_{r}}+(B+\frac{C}{r^{2}})\sin\theta\overrightarrow{e_{\theta}}$ (F7)

$\overrightarrow{H}_{3}=-\nabla\phi_{m3}=(-D+\frac{E}{r^{2}})\cos\theta\overrightarrow{e_{r}}+(D+\frac{E}{r^{2}})\sin\theta\overrightarrow{e_{\theta}}$ (F8)

$\overrightarrow{H}_{4}=-\nabla\phi_{m4}=-F\cos\theta\overrightarrow{e_{r}}+F\sin\theta\overrightarrow{e_{\theta}}$ (F9)

Due to the magneto-electric response of the CAM, the electric potential in the four regions can be induced,

$\phi_{e1}=(A_{0}r+\frac{A_{1}}{r})\cos\theta$ (F10)

$\phi_{e2}=(B_{1}r+\frac{C_{1}}{r})\cos\theta$ (F11)

$\phi_{e3}=(D_{1}r+\frac{E_{1}}{r})\cos\theta$ (F12)

$\phi_{e4}=F_{1}r\cos\theta$ (F13)

where $\phi_{e1}$, $\phi_{e2}$, $\phi_{e3}$ and $\phi_{e4}$ are the electric potential in the four regions, and $A_{1}$, $B_{1}$, $C_{1}$, $D_{1}$, $E_{1}$ and $F_{1}$ are unknown coefficients. Because of no physics electric field in region I, so $A_{0}=0$. The relation between the electric field and the electric potential is $\overrightarrow{E}=-\nabla\phi_{e}$, where $\overrightarrow{E}$ is the electric field, the electric field in the four regions can be written as

$\overrightarrow{E}_{1}=-\nabla\phi_{e1}=\frac{A_{1}}{r^{2}}\cos\theta\overrightarrow{e_{r}}+\frac{A_{1}}{r^{2}}\sin\theta\overrightarrow{e_{\theta}}$ (F14)

$\overrightarrow{E}_{2}=-\nabla\phi_{e2}=(-B_{1}+\frac{C_{1}}{r^{2}})\cos\theta\overrightarrow{e_{r}}+(B_{1}+\frac{C_{1}}{r^{2}})\sin\theta\overrightarrow{e_{\theta}}$ (F15)

$\overrightarrow{E}_{3}=-\nabla\phi_{e3}=(-D_{1}+\frac{E_{1}}{r^{2}})\cos\theta\overrightarrow{e_{r}}+(D_{1}+\frac{E_{1}}{r^{2}})\sin\theta\overrightarrow{e_{\theta}}$ (F16)

$\overrightarrow{E}_{4}=-\nabla\phi_{e4}=-F_{1}\cos\theta\overrightarrow{e_{r}}+F_{1}\sin\theta\overrightarrow{e_{\theta}}$ (F17)

The magnetic field is continuous in the tangential direction

$-H_{0}+\frac{A}{a^{2}}=B+\frac{C}{a^{2}}$ (F18)

$D+\frac{E}{b^{2}}=B+\frac{C}{b^{2}}$ (F19)

$D+\frac{E}{c^{2}}=F$ (F20)

The electric field is continuous in the tangential direction

$\frac{A_{1}}{a^{2}}=B_{1}+\frac{C_{1}}{a^{2}}$ (F21)

$D_{1}+\frac{E_{1}}{b^{2}}=B_{1}+\frac{C_{1}}{b^{2}}$ (F22)

$D_{1}+\frac{E_{1}}{c^{2}}=F_{1}$ (F23)

The magnetic flux density is continuous in the normal direction, combining Eq. (A2) and Eq. (F5), we obtain

$\mu_{0}\mu_{1}(H_{0}+\frac{A}{a^{2}})=\mu_{0}\mu_{2}(-B+\frac{C}{a^{2}})+\mu_{0}M_{2}-\mu_{2}\sqrt{\varepsilon_{0}\mu_{0}}\frac{\alpha\theta_{2}}{\pi}(-B_{1}+\frac{C_{1}}{a^{2}})$ (F24)

$\mu_{0}\mu_{2}(-B+\frac{C}{b^{2}})+\mu_{0}M_{2}-\mu_{2}\sqrt{\varepsilon_{0}\mu_{0}}\frac{\alpha\theta_{2}}{\pi}(-B_{1}+\frac{C_{1}}{b^{2}})$ $=\mu_{0}\mu_{3}(-D+\frac{E}{b^{2}})+\mu_{0}M_{3}-\mu_{3}\sqrt{\varepsilon_{0}\mu_{0}}\frac{\alpha\theta_{3}}{\pi}(-D_{1}+\frac{E_{1}}{b^{2}})$ (F25)

$\mu_{0}\mu_{3}(-D+\frac{E}{c^{2}})+\mu_{0}M_{3}-\mu_{3}\sqrt{\varepsilon_{0}\mu_{0}}\frac{\alpha\theta_{3}}{\pi}(-D_{1}+\frac{E_{1}}{c^{2}})=-\mu_{0}\mu_{4}F$ (F26)

Noting that, the normal magnetization intensity $\overrightarrow{M}_{r}=\overrightarrow{M}\cdot\overrightarrow{e}_{r}=M\cos\theta$. The electric displacement is continuous in the normal direction, according to Eq. (A1), we obtain

$\varepsilon_{0}\varepsilon_{1}\frac{A_{1}}{a^{2}}=\varepsilon_{0}(\varepsilon_{2}+\mu_{2}\frac{\alpha^{2}\theta_{2}^{2}}{\pi^{2}})(-B_{1}+\frac{C_{1}}{a^{2}})-\mu_{2}\sqrt{\varepsilon_{0}\mu_{0}}\frac{\alpha\theta_{2}}{\pi}(-B+\frac{C}{a^{2}})$ (F27)

$\varepsilon_{0}(\varepsilon_{2}+\mu_{2}\frac{\alpha^{2}\theta_{2}^{2}}{\pi^{2}})(-B_{1}+\frac{C_{1}}{b^{2}})-\mu_{2}\sqrt{\varepsilon_{0}\mu_{0}}\frac{\alpha\theta_{2}}{\pi}(-B+\frac{C}{b^{2}})$ $=\varepsilon_{0}(\varepsilon_{3}+\mu_{3}\frac{\alpha^{2}\theta_{3}^{2}}{\pi^{2}})(-D_{1}+\frac{E_{1}}{b^{2}})-\mu_{3}\sqrt{\varepsilon_{0}\mu_{0}}\frac{\alpha\theta_{3}}{\pi}(-D+\frac{E}{b^{2}})$ (F28)

$\varepsilon_{0}(\varepsilon_{3}+\mu_{3}\frac{\alpha^{2}\theta_{3}^{2}}{\pi^{2}})(-D_{1}+\frac{E_{1}}{c^{2}})-\mu_{3}\sqrt{\varepsilon_{0}\mu_{0}}\frac{\alpha\theta_{3}}{\pi}(-D+\frac{E}{c^{2}})=-\varepsilon_{0}\varepsilon_{4}F_{1}$ (F29)

When $A=0$, the magnetic field is still $H_{0}$ in region I, when $F=0$, the magnetic field vanishes in region IV, when $A_{1}$, $B_{1}$, $C_{1}$, $D_{1}$, $E_{1}$ and $F_{1}$ are non-zero, the electric field is induced by the applied magnetic field.


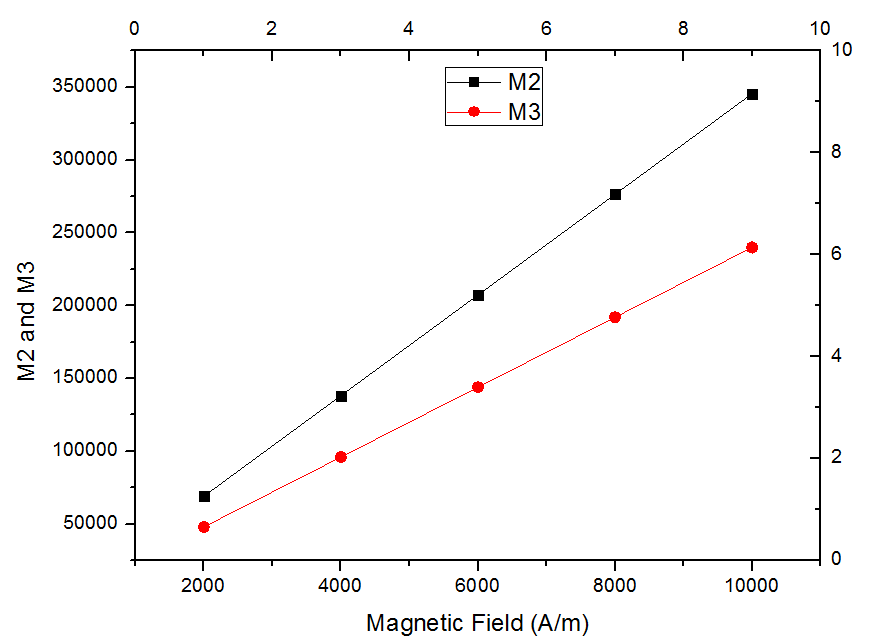


Fig. F2. The relations between the intrinsic magnetization intensity and the applied magnetic field $H_{0}$


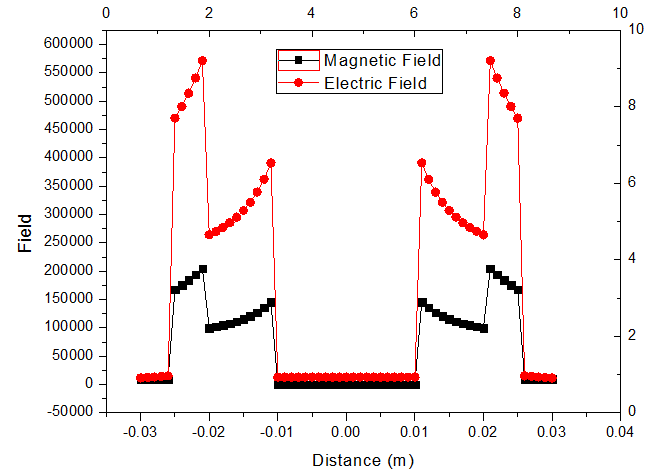


Fig. F3. The distributions of the magnetic field and electric field in the center line ($\text{y}=0$, $\text{z}=0$).

The bi-layer CAM cylinder doped with different NdFeB (for example, the intrinsic magnetization intensity of the N35 is 1.17 - 1.22 T, and the N50 is 1.40 - 1.45 T) can cloak different applied magnetic field, Fig. F2 shows the relations between the intrinsic magnetization intensity and the applied magnetic field $H_{0}$, the magneto-electric polarizability $\theta_{2}=\pi$, $\theta_{3}=\pi$; the other parameters are $\varepsilon_{1}=1$, $\varepsilon_{2}=2$, $\varepsilon_{3}=1.5$, $\varepsilon_{4}=1$, $\mu_{1}=1$, $\mu_{2}=2$, $\mu_{3}=1.5$, $\mu_{4}=1$; the fine-structure constant $\alpha=1/137$, the permittivity and permeability of the free space $\varepsilon_{0}=8.85\times10^{12}F/m$, $\mu_{0}=4\pi\times10^{-7}H/m$, respectively, the size parameter $a=0.0\text{25m}$, $b=0.02\text{0m}$, $c=0.01\text{0m}$. The different intrinsic magnetization intensity can cloak different applied magnetic field, for example, when the intrinsic magnetization intensity are $M_{2}=3.455\times10^{5}A/m$ are $M_{3}=-2.400\times10^{5}A/m$, we can cloak the applied magnetic field $H_{0}=10000A/m$; when the intrinsic magnetization intensity are $M_{2}=2.073\times10^{5}A/m$ and $M_{3}=-1.440\times10^{5}A/m$, we can cloak the applied magnetic field $H_{0}=6000A/m$.

Fig. F3 shows the distributions of the magnetic field and the electric field in the center line ($\text{y}=0$, $\text{z}=0$), the black lines stand for the magnetic field in the center line, and the red line stands for the electric field in the center line, when $r>0.025m$, the magnetic field is still $H_{0}=10000A/m$, yet the induced electric field varies with space; when $0.020m<r<0.025m$, the magnetic field and the induced electric field both vary with space; when $0.010m<r<0.020m$, the magnetic field and the induced electric field also vary with space; when $r<0.010m$, the magnetic field is $H_{4}=0$, $E_{4}=13022V/m$. Namely, the bi-layer CAM cylinder cloaked applied magnetic field and induced electric field.

**Reference:**

S1. Gömöry, F. et al. Experimental realization of a magnetic cloak. *Science* **335**, 1466–1468 (2012).

S2. Zeng, L. W., Song, R. X., & Zeng, J. Inducing magneto-electric response in topological insulator. *Journal of Magnetism and Magnetic Materials*, **328**, 26-30 (2013).
